# Dependence of PAX3-FOXO1 chromatin occupancy on ETS1 at important disease-promoting genes exposes new targetable vulnerability in Fusion-Positive Rhabdomyosarcoma

**DOI:** 10.1038/s41388-024-03201-2

**Published:** 2024-10-24

**Authors:** Joseph Hsieh, Etienne P. Danis, Charles R. Owens, Janet K. Parrish, Nathan L. Nowling, Arthur R. Wolin, Stephen Connor Purdy, Sheera R. Rosenbaum, Atma M. Ivancevic, Edward B. Chuong, Heide L. Ford, Paul Jedlicka

**Affiliations:** 1 Medical Scientist Training Program, University of Colorado Anschutz Medical Campus (UC-AMC), Aurora, CO USA; 2 Cancer Biology Graduate Training Program, UC-AMC, Aurora, CO USA; 3 Department of Pathology, UC-AMC, Aurora, CO USA; 4 Department of Biomedical Informatics, UC-AMC, Aurora, CO USA; 5 University of Colorado Cancer Center, UC-AMC, Aurora, CO USA; 6 Department of Pharmacology, UC-AMC, Aurora, CO USA; 7 Molecular Biology Graduate Training Program, UC-AMC, Aurora, CO USA; 8 Department of Molecular, Cellular, and Developmental Biology and BioFrontiers Institute, University of Colorado Boulder, Boulder, CO USA

**Keywords:** Sarcoma, Paediatric cancer, Epigenetics

## Abstract

Rhabdomyosarcoma (RMS), a malignancy of impaired myogenic differentiation, is the most common soft tissue pediatric cancer. PAX3-FOXO1 oncofusions drive the majority of the clinically more aggressive fusion-positive rhabdomyosarcoma (FP-RMS). Recent studies have established an epigenetic basis for PAX3-FOXO1-driven oncogenic processes. However, details of PAX3-FOXO1 epigenetic mechanisms, including interactions with, and dependence on, other chromatin and transcription factors, are incompletely understood. We previously identified a novel disease-promoting epigenetic axis in RMS, involving the histone demethylase KDM3A and the ETS1 transcription factor, and demonstrated that this epigenetic axis interfaces with PAX3-FOXO1 both phenotypically and transcriptomically, including co-regulation of biological processes and genes important to FP-RMS progression. In this study, we demonstrate that KDM3A and ETS1 colocalize with PAX3-FOXO1 to enhancers of important disease-promoting genes in FP-RMS, including FGF8, IL4R, and MEST, as well as PODXL, which we define herein as a new FP-RMS-promoting gene. We show that ETS1, which is induced by both PAX3-FOXO1 and KDM3A, exists in complex with PAX3-FOXO1, and augments PAX3-FOXO1 chromatin occupancy. We further show that the PAX3-FOXO1/ETS1 complex can be disrupted by the clinically relevant small molecule inhibitor YK-4-279. YK-4-279 displaces PAX3-FOXO1 from chromatin and interferes with PAX3-FOXO1-dependent gene regulation, resulting in potent inhibition of growth and invasive properties in FP-RMS, along with downregulation of FGF8, IL4R, MEST and PODXL expression. We additionally show that, in some FP-RMS, KDM3A also increases PAX3-FOXO1 levels. Together, our studies illuminate mechanisms of action of the KDM3A/ETS1 regulatory module, and reveal novel targetable mechanisms of PAX3-FOXO1 chromatin complex regulation, in FP-RMS.

## Introduction

Rhabdomyosarcoma (RMS), a malignancy of impaired myogenic differentiation, is the most common soft tissue pediatric cancer [1]. Molecularly, RMS can be classified by oncofusion status into fusion-negative RMS (FN-RMS) and fusion-positive RMS (FP-RMS) with the fusion status conferring critical clinical prognostic value [2–4]. FN-RMS typically shows embryonal RMS (ERMS) histology, and portends better outcomes (>70% 5-year survival with appropriate care). Conversely, FP-RMS typically shows alveolar RMS (ARMS) histology, and has much less favorable outcomes; patients with FP-RMS have a 39% 5-year overall survival, which drops to <20% for patients with metastatic or recurrent diseases [5–7]. The current treatment paradigm for FP-RMS, a high-risk disease by definition, consists of high-dose multi-agent chemotherapy, in combination with surgery and radiation [8]. Despite this aggressive treatment, and associated adverse effects of therapy, many FP-RMS patients still suffer dismal outcomes, demonstrating a dire need for increased understanding of FP-RMS pathogenesis and identification of better therapies.

PAX3-FOXO1 (P3F) is the driver oncofusion in 70% of FP-RMS, with a less aggressive PAX7-FOXO1 oncofusion making up most of the remaining FP-RMS cases [6]. The PAX3-FOXO1 oncofusion stems from chromosomal translocations fusing the amino terminus of PAX3, with an intact DNA-binding domain, to the carboxy terminus of FOXO1, with an intact transactivation domain. This fusion yields the neomorphic transcription factor PAX3-FOXO1 that aberrantly regulates gene expression to promote cell proliferation, impair myogenic differentiation, and promote metastatic properties such as invasion. Studies investigating PAX3-FOXO1 molecular action have revealed important epigenetic mechanisms in the disease-driving functions of PAX3-FOXO1, including activation of enhancers and so-called ‘super-enhancers’, interactions with chromatin factors, and hijacking of myogenic transcription factor networks [9–12]. Since the PAX3-FOXO1 oncofusion itself presents a difficult therapeutic target, understanding how P3F functions in conjunction with other chromatin and transcription factors presents important alternative mechanisms to treat FP-RMS.

Our prior studies identified a novel disease-promoting epigenetic axis in the pediatric sarcomas Ewing Sarcoma, FN-RMS, and FP-RMS, involving the chromatin factor KDM3A, a member of the Jumonji-domain histone demethylase family, and the transcription factor ETS1, a member of the ETS transcription factor family [13–15]. In all three diseases, ETS1 is downstream of, and upregulated by, KDM3A. Moreover, in FP-RMS, ETS1 is also downstream of, and upregulated by, PAX3-FOXO1 [14]. In FP-RMS, KDM3A and ETS1 each promote cell growth and invasive properties [14, 15], similar to PAX3-FOXO1 [16], and strongly mirror PAX3-FOXO1 transcriptome regulation, including upregulation of known genes important to disease progression [14, 17–19]. In the present study, we sought to further understand how KDM3A, ETS1, and PAX3-FOXO1 collaborate mechanistically to drive FP-RMS oncogenic properties.

## Results

### KDM3A, ETS1, and PAX3-FOXO1 co-localize to enhancers of important disease-promoting genes in FP-RMS

**Fig. 1:**
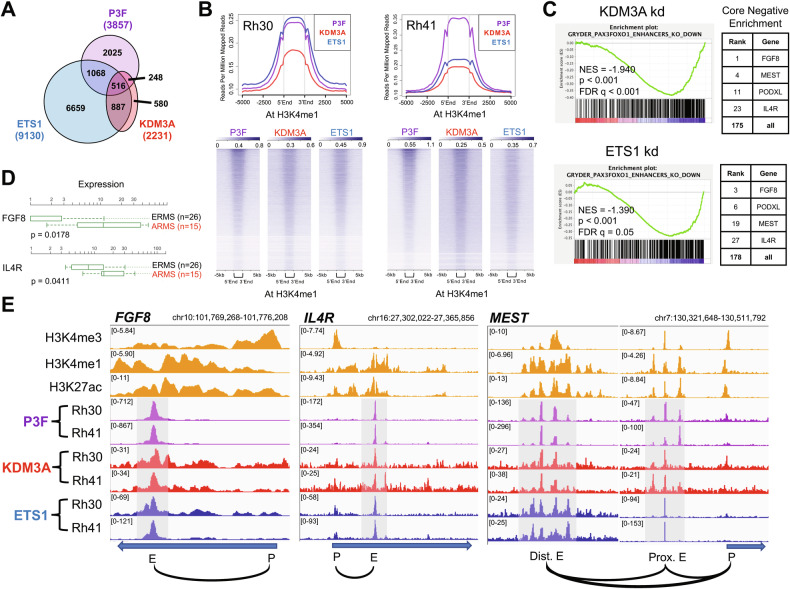
KDM3A and ETS1 co-localize with PAX3-FOXO1 (P3F) to enhancers of important disease-promoting genes in FP-RMS. **A** Overlap (Venn) analysis of CUT&RUN cistrome peaks, common to both Rh30 and Rh41 FP-RMS cells, for KDM3A, ETS1, and PAX3-FOXO1. **B** Genomic distribution of KDM3A and ETS1, relative to PAX3-FOXO1, at FP-RMS enhancers (marked by H3K4me1; data from [10], via CistromeDB (http://cistrome.org)), as determined using ngs.plot analysis of our CUT&RUN data. Global distribution profiles (top panels) and corresponding heat maps (bottom panels). **C** Downregulation of PAX3-FOXO1 enhancer-activated genes, as defined in [10], upon KDM3A (top) and ETS1 (bottom) knockdown, as determined by Gene Set Enrichment Analysis (GSEA) of our KDM3A and ETS1 transcriptome data from [14]. NES: Normalized Enrichment Score; p: nominal p-value; FDR q: False Discovery Rate (q-value)). Right: selected top-ranked genes contributing to GSEA core negative enrichment. **D** Gene expression levels of FGF8 and IL4R in ERMS (FN-RMS) and ARMS (FP-RMS) patient tumors (data from St. Jude Children’s Research Hospital PeCan database; pecan.stjude.cloud); p-value from two-tailed Welch’s t-test (unpaired t-test with unequal variance). **E** PAX3-FOXO1, KDM3A, and ETS1 CUT&RUN cistrome tracks at the FGF8, IL4R, and MEST loci in Rh30 and Rh41 cells, as visualized using the Integrated Genomics Viewer (IGV; FP-RMS H3K4me3, H3K4me1, and H3K27ac reference tracks are from [10], via CistromeDB). Blue arrows depict transcript direction. Black curved lines denote chromatin regional associations (Rh30 Hi-C data from ENCODE3, generated by Dekker Laboratory). “P”: promoter; “E”: enhancer; “Prox.”: proximal (relative to transcription start site); “Dist.”: distal.

Our previous studies identified a novel KDM3A/ETS1 epigenetic axis that cooperates with PAX3-FOXO1 phenotypically and transcriptomically to drive FP-RMS [14, 15]. In order to understand how KDM3A, ETS1, and PAX3-FOXO1 interact at the chromatin level to regulate FP-RMS disease-promoting gene expression, we performed CUT&RUN cistrome profiling in patient-derived Rh30 and Rh41 FP-RMS cells. For KDM3A and ETS1, our CUT&RUN analysis identified 2231 and 9130 chromatin localization sites, respectively, common to both cell lines (Fig. 1A). Integration with our previous transcriptome data for KDM3A and ETS1 in the same cell lines [14] confirmed regulatory control of genes related to cancer growth and metastasis (Fig. S1). In parallel, we also performed CUT&RUN cistrome profiling for PAX3-FOXO1, using C-terminal FOXO1 antibody, an approach rigorously validated for PAX3-FOXO1-specific cistrome profiling in FP-RMS [12], and also used by others [20]. Our CUT&RUN revealed 3857 PAX3-FOXO1 sites, common to both Rh30 and Rh41 cells (Fig. 1A and S2).

Consistent with coregulation observed in our prior transcriptome studies [14]: KDM3A and ETS1 each showed genomic colocalization with PAX3-FOXO1, as well as with one another, in FP-RMS (Fig. 1A); all three factors localized to active regulatory elements (marked by H3K27ac), including promoters (marked by H3K4me3) and enhancers (marked by H3K4me1) (Fig. 1B and S3). Enhancer-dependent mechanisms have previously been shown to play a particularly important role in PAX3-FOXO1-dependent gene expression in FP-RMS [10]. Notably, KDM3A and ETS1 knockdown [14] each resulted in reduced expression of PAX3-FOXO1 enhancer-activated genes in FP-RMS (Fig. 1C). Our prior transcriptome studies showed that KDM3A and ETS1 both coregulate the PAX3-FOXO1 super-enhancer-activated genes FGF8, IL4R, and MEST [10, 14]. Notably, all these genes promote growth and metastatic properties important to FP-RMS progression [14, 17–19]. Additionally, FGF8 and IL4R are overexpressed in FP-RMS relative to FN-RMS (Fig. 1D), and thus constitute potentially important mediators of FP-RMS aggressive biological properties. Of PAX3-FOXO1 enhancer-activated genes, FGF8, IL4R, and MEST were among the most strongly down-regulated genes following both KDM3A and ETS1 knockdown (Fig. 1C). Moreover, their enhancer elements were strongly co-bound by KDM3A and ETS1, along with PAX3-FOXO1 (Fig. 1E). Taken together, our data indicate that KDM3A and ETS1 directly coregulate, with PAX3-FOXO1, important disease-promoting genes in FP-RMS, and likely do so at least in part through enhancer mechanisms.

### PODXL, upregulated by PAX3-FOXO1, KDM3A and ETS1, is a novel promoter of aggressive properties in FP-RMS

**Fig. 2:**
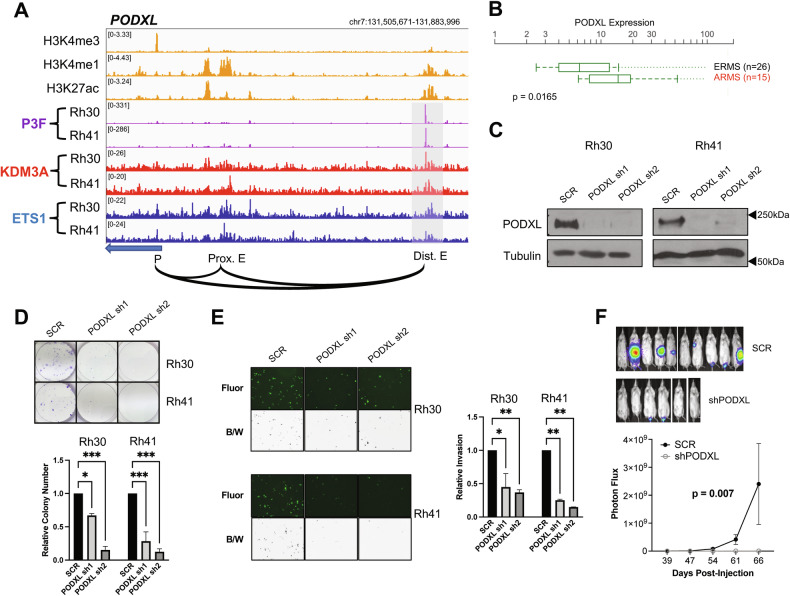
PODXL, regulated by PAX3-FOXO1, KDM3A, and ETS1, promotes growth, invasion, and metastasis in FP-RMS. **A** PAX3-FOXO1, KDM3A, and ETS1 cistrome tracks, and reference FP-RMS histone mark tracks (H3K4me3, H3K4me1, and H3K27ac) at the PODXL genomic locus, plotted as in Fig. 1. **B** PODXL expression in ERMS (FN-RMS) and ARMS (FP-RMS) patient tumors (data from St. Jude Children’s Research Hospital PeCan database; pecan.stjude.cloud); p-value from two-tailed Welch’s t-test. **C** PODXL shRNA-medicated knockdown in Rh30 and Rh41 cells, as determined by immunoblotting, with tubulin as loading control. SCR: Scrambled shRNA negative control. **D** Top: Representative images from a colony formation assay in control (SCR), and PODXL knock-down, Rh30 and Rh41 cells. Bottom: Quantification of colony formation assay data, plotted as mean and standard error of the mean (s.e.m.), from two independent experiments, each performed in triplicate, with the control (SCR) set to 1. *p < 0.05; ***p < 0.001 (from one-way analysis of variance (ANOVA) with multiple comparisons). **E** Left: Representative images from a transendothelial invasion assay in control (SCR), and PODXL knock-down, Rh30 and Rh41 cells. Data shown both as fluorescence images (Calcein AM dye-labeled cells; “Fluor”), and as pseudo-colored images (black on a white background; “B/W”), for greater ease of visualization. Right: Quantification of invasion data, plotted as mean and s.e.m. from two independent experiments, each performed in triplicate, with the control (SCR) set to 1. *p < 0.05; **p < 0.01 (from one-way ANOVA with multiple comparisons). **F** Effect of PODXL depletion on metastasis in a tail vein injection model. Scrambled control (SCR) or shPODXL (sh1) Rh30 cells, each additionally expressing a luciferase reporter, were injected into the tail vein of NOD-SCID/gamma mice (n = 10 for SCR; n = 6 for shPODXL). Metastasis development was monitored using IVIS imaging following administration of luciferin. Top: Representative IVIS images at end of experiment. Bottom: IVIS quantification data (mean and s.e.m. of photon flux over time course of experiment); p-value from 2-way ANOVA with repeated measures.

In our transcriptome and cistrome analyses, we noted the gene PODXL as a PAX3-FOXO1 super-enhancer-activated [10], and KDM3A and ETS1 coregulated [14], gene. Indeed, along with FGF8, IL4R, and MEST, PODXL was one of the most strongly KDM3A and ETS1-regulated genes among PAX3-FOXO1 enhancer-activated genes (Fig. 1C). At the cistrome level, KDM3A and ETS1 showed colocalization with PAX3-FOXO1 at the PODXL gene locus, including both the proximal and distal enhancers, the latter with particularly high PAX3-FOXO1 levels (Fig. 2A; specificity of KDM3A and ETS1 localization confirmed with factor knockdown (Fig. S4)). PODXL codes for a transmembrane glycoprotein that has been previously implicated in endothelial cell adhesion and transvasation during cancer metastasis [21]. Notably, we previously showed that KDM3A and ETS1 both promote the metastatic property of transendothelial invasion in FP-RMS [14, 15]. Thus, PODXL could be an important mediator of these effects. Furthermore, similar to FGF8 and IL4R, both known important metastasis-promoters in FP-RMS, PODXL shows strong overexpression in FP-RMS, relative to FN-RMS (Fig. 2B). We were thus interested whether PODXL, a factor not previously studied in RMS, could be an important new promoter of aggressive properties in FP-RMS, downstream of PAX3-FOXO1, KDM3A, and ETS1.

To evaluate the role of PODXL in FP-RMS, we employed loss-of-function studies and phenotypic analyses of growth, invasion, and metastasis in FP-RMS cells, analogous to our prior studies of KDM3A and ETS1 [14, 15]. Upon shRNA-mediated knockdown of PODXL expression (Fig. 2C), we observed reduction of both colony growth and transendothelial invasion in vitro (Fig. 2D, E). Moreover, in the tail vein injection model of post-intravasation metastasis, PODXL knockdown significantly decreased metastatic seeding/outgrowth (Fig. 2F). Thus, the transmembrane protein PODXL, strongly upregulated in FP-RMS by PAX3-FOXO1, KDM3A and ETS1, is a novel promoter of FP-RMS aggressive properties.

### BRG1 does not promote invasive properties in FP-RMS

BRG1, the ATPase subunit of the BAF chromatin remodeling complex, has been shown to cooperate with KDM3A and ETS1 on chromatin [22, 23], and to promote invasive and metastatic properties in other cancers [24, 25]. Furthermore, BRG1 has recently been shown to exist in a complex with PAX3-FOXO1 and to promote cell proliferation, and inhibit differentiation, in FP-RMS [26, 27]. We were thus interested in further understanding the role of BRG1 in FP-RMS, both with respect to KDM3A and ETS1 cistromes, and aggressive phenotypic properties. CUT&RUN cistrome profiling revealed broad distribution of BRG1 across the FP-RMS genome, including 20069 sites common to Rh30 and Rh41 FP-RMS cells (Fig. S5A). Peak overlap analysis showed BRG1 to be present at the vast majority of KDM3A and ETS1, as well as PAX3-FOXO1, bound loci, including at enhancers of FGF8, IL4R, MEST, and PODXL (Fig. S5A, B). Based on these findings, we hypothesized that BRG1 co-contributes to aggressive properties in FP-RMS. Analogous to our other studies, we thus stably depleted BRG1 expression in Rh30 and Rh41 FP-RMS using shRNA (Fig. S6A), and examined the effects on growth and invasive properties. These analyses confirmed that BRG1 promotes growth properties in FP-RMS (Fig. S6B), concordant with prior findings [26, 27]. Interestingly, however, in both cell lines, BRG1 knockdown did not inhibit cell migration, matrix invasion, or transendothelial invasion (Fig. S6C–E); in fact, in some experiments, BRG1 knockdown showed a trend toward increased migration/invasion. Thus, in contrast to KDM3A and ETS1 [14, 15], as well as PAX3-FOXO1 itself [16], BRG1 does not promote invasive properties in FP-RMS.

### ETS1 promotes PAX3-FOXO1 chromatin occupancy

Given their colocalization at important disease-promoting genes (Fig. 1), and convergent phenotypic properties (from our prior studies [14, 15]), we further investigated the mechanistic relationships of KDM3A and ETS1 to PAX3-FOXO1 on chromatin. Specifically, we wondered whether KDM3A and/or ETS1 might act in part by regulating PAX3-FOXO1 chromatin occupancy. To address this question, we used CUT&RUN to compare cistrome profiles in control and factor-depleted cells. Control and factor-depleted samples were normalized to one another using total human reads, and analyses focused on genomic loci co-bound by PAX3-FOXO1, KDM3A and ETS1 (516 ‘P/K/E’ sites; as defined in Fig. 1A). In the same experiments, we additionally examined effects on overall factor protein levels, as these would be expected to impact any changes in factor levels on chromatin.

**Fig. 3:**
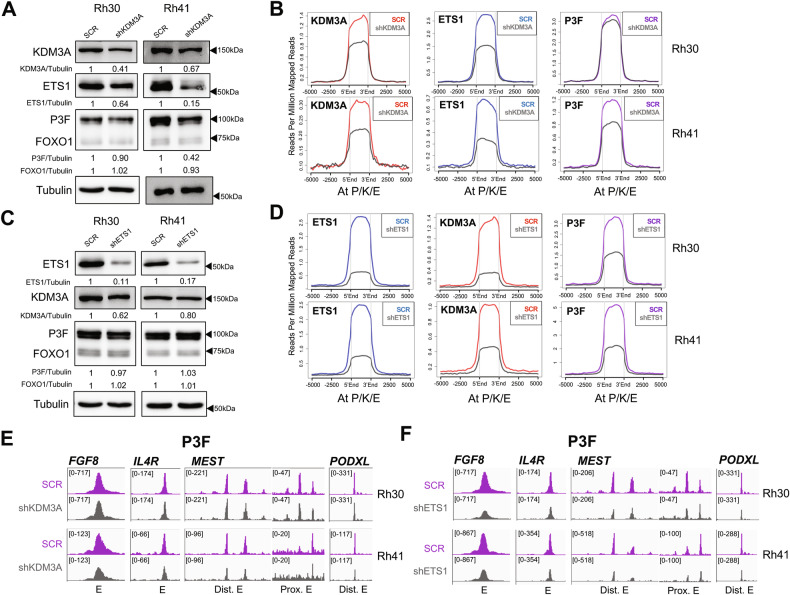
Regulation of PAX3-FOXO1 levels and chromatin occupancy by KDM3A and ETS1. **A** Effects of KDM3A knockdown on KDM3A, PAX3-FOXO1, FOXO1 and ETS1 total protein levels, as determined by immunoblotting and quantified by densitometry (Factor/Tubulin), in Rh30 and Rh41 cells. **B** Relative levels of KDM3A, ETS1, and PAX3-FOXO1, localizing to PAX3-FOXO1, KDM3A, and ETS1 co-bound sites (“P/K/E”, as defined in Fig. 1A), in control (SCR) and KDM3A knockdown (shKDM3A) cells, as determined by CUT&RUN cistrome profiling with internal normalization, in Rh30 and Rh41 cells; data plotted using ngs.plot. **C** Effects of ETS1 knock-down on ETS1, KDM3A, PAX3-FOXO1 and FOXO1 total protein levels, as determined by immunoblotting and quantified by densitometry (Factor/Tubulin). **D** Relative levels of ETS1, KDM3A, and PAX3-FOXO1 localizing to PAX3-FOXO1, KDM3A, and ETS1 co-bound sites (“P/K/E”), in control (SCR) and ETS1 knockdown (shETS1) cells, as determined by CUT&RUN cistrome profiling with internal normalization, plotted as in **B**, using ngs.plot. **E** CUT&RUN data for PAX3-FOXO1 from control and KDM3A knockdown cells (same data as in **B**), visualized at enhancer regulatory elements of the FGF8, IL4R, MEST, and PODXL genes, using IGV. Enhancer regions shown correspond to gray-shaded regions from Figs. 1E and 2A. “E”: enhancer; “Dist.”: distal; “Prox.”: proximal. **F** CUT&RUN data for PAX3-FOXO1 from control and ETS1 knockdown cells (same data as in **D**), visualized at FGF8, IL4R, MEST, and PODXL enhancers, as in **E**.

KDM3A knockdown and ETS1 knockdown, in both Rh30 and Rh41 cells (Fig. 3A, C), each resulted in reduced levels of the respective factors on chromatin (Fig. 3B, D), confirming our cistrome profile normalization strategy. KDM3A knockdown in both cell lines also resulted in reduced total ETS1 protein levels (Fig. 3A), confirming our prior observations of ETS1 being under positive regulatory control by KDM3A [14]. Accordingly, this also translated into reduced levels of ETS1 on chromatin in both cell lines (Fig. 3B). In Rh41 cells, but not Rh30 cells, KDM3A knockdown reduced PAX3-FOXO1 levels by approximately 50% (Fig. 3A; without affecting levels of FOXO1). Accordingly, this also translated into a reduction of PAX3-FOXO1 levels on chromatin in Rh41 cells, but not Rh30 cells (Fig. 3B, E). The lack of an effect on PAX3-FOXO1 chromatin levels in Rh30 cells suggests that KDM3A does not exert control over PAX3-FOXO1 chromatin occupancy independent of total protein level effects.

ETS1 knockdown reduced total KDM3A protein levels approximately 40% in Rh30 cells and 20% in Rh41 cells (Fig. 3C). At the chromatin level, ETS1 knockdown resulted in well over 50% reduction in KDM3A in both cell lines (Fig. 3D). Thus, ETS1 exerts positive regulatory control over KDM3A protein levels, and, given the greater magnitude of the chromatin effect, may also promote KDM3A chromatin occupancy through more direct mechanisms. ETS1 depletion also resulted in a robust, 50% or greater, decrease in PAX3-FOXO1 chromatin occupancy in both cell lines (Fig. 3D, F). Strikingly, this was unaccompanied by any changes in total PAX3-FOXO1 protein levels (or FOXO1 protein levels; Fig. 3C). Thus, ETS1 positively controls PAX3-FOXO1 chromatin occupancy through a more direct, chromatin-level, mechanism, independent of effects on total protein.

Given the different effects of KDM3A knockdown on PAX3-FOXO1 levels in Rh30 and Rh41 cells, we examined additional FP-RMS cell lines. KDM3A knockdown robustly reduced PAX3-FOXO1 levels in Rh4 cells, but not Rh5 cells (Fig. S7). Similar to our previous findings in Rh30 and Rh41 cells [14], KDM3A knockdown robustly reduced ETS1 levels in both Rh4 and Rh5 cells (Fig. S7). Thus, KDM3A consistently upregulates ETS1 in FP-RMS. Moreover, in some FP-RMS, KDM3A also positively controls PAX3-FOXO1 levels.

### ETS1 and PAX3-FOXO1 exist in a shared complex

**Fig. 4:**
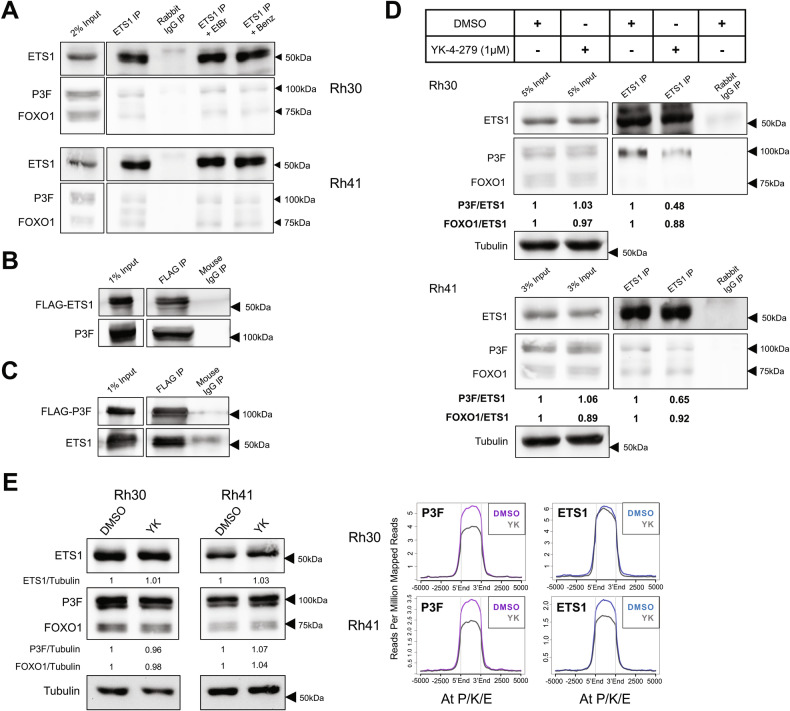
ETS1 interactions with PAX3-FOXO1, and effects of YK-4-279. **A** Immunoblot showing co-immunoprecipitation (co-IP) of PAX3-FOXO1 (and FOXO1) with ETS1 in Rh30 and Rh41 cells, with ETS1 antibody (lane 2), but not negative control antibody (rabbit IgG, lane 3). Lanes 4 and 5: same immunoprecipitation (IP) reactions as in lane 2, but also with Ethidium Bromide (EtBr; lane 4), or Benzonase (Benz; lane 5), to disrupt protein-nucleic acid interactions. 2% input also shown (lane 1). **B** Immunoblot showing co-IP of PAX3-FOXO1 with FLAG-tagged ETS1 upon ectopic expression of both proteins in 293FT cells. Lane 1: 1% input. Lanes 2 and 3: IP with FLAG or negative control antibody (mouse IgG), respectively. **C** Immunoblot showing co-IP of ETS1 with FLAG-tagged PAX3-FOXO1 upon ectopic expression of both proteins in 293FT cells. Lane 1: 1% input. Lanes 2 and 3: IP with FLAG or negative control antibody (mouse IgG), respectively. **D** Effects of YK-4-279 treatment on co-IP of PAX3-FOXO1 (and FOXO1) with ETS1 from Rh30 and Rh41 cells. Cells were pre-treated for 16 hours with 1 µM YK-4-279 or vehicle (DMSO), and IP was carried out as in panel A, but also with inclusion of 1 µM YK-4-279 or DMSO in the IP reaction. Relative recovery of PAX3-FOXO1 (P3F; and FOXO1), normalized to ETS1 (“P3F/ETS1” and “FOXO1/ETS1”), was determined by densitometry (lanes 3 and 4). Relative ratios of P3F (and FOXO1) to ETS1 in corresponding input samples are also shown (lanes 1 and 2), as is IP with negative control antibody (lane 5). **E** Effects of YK-4-279 treatment on PAX3-FOXO1 and ETS1 chromatin occupancy. Rh30 and Rh41 cells were treated with 1 µM YK-4-279 or vehicle (DMSO) for 5 hours prior to CUT&RUN cistrome profiling. Left: Immunoblot showing ETS1, PAX3-FOXO1 (P3F) and FOXO1 total protein levels in vehicle and YK-4-279 treated cells (5 hours), quantified by densitometry (Factor/Tubulin). Right: Relative levels of ETS1 (top panels) and PAX3-FOXO1 (bottom panels), localizing to PAX3-FOXO1, KDM3A, and ETS1 co-bound sites (“P/K/E”, as defined in Fig. 1A), in control (DMSO) and YK-4-279-treated (YK) cells, as determined by CUT&RUN cistrome profiling with internal normalization; data plotted using ngs.plot.

Following up on the finding that ETS1 promotes PAX3-FOXO1 chromatin occupancy (Fig. 3), we asked whether these effects could involve protein-protein interactions. We first examined whether endogenous ETS1 and PAX3-FOXO1 proteins exist in a contemporaneous complex, using co-immunoprecipitation studies in FP-RMS cells. In both Rh30 and Rh41 cells, immunoprecipitation with ETS1 antibody, but not non-specific antibody control (IgG), co-immunoprecipitated PAX3-FOXO1 (Fig. 4A), indicating that the two proteins exist in a shared complex. Furthermore, treatment with either Ethidium Bromide or Benzonase, both disruptors of protein-nucleic acid interactions, did not interfere with PAX3-FOXO1 co-immunoprecipitation (Fig. 4A), indicating that the coexistence of ETS1 and PAX3-FOXO1 in this shared complex is independent of DNA or other nucleic acids. In the same experiments, ETS1 also co-immunoprecipitated FOXO1 (Fig. 4A), a known interaction from other biological contexts [28, 29].

Next, we examined whether ETS1 and PAX3-FOXO1 can interact in a heterologous context, using ectopic protein expression in 293FT cells. Ectopic expression of FLAG-tagged ETS1 and (untagged) PAX3-FOXO1, followed by immunoprecipitation with FLAG antibody, co-immunoprecipitated PAX3-FOXO1 (Fig. 4B). In the reciprocal experiment, ectopic expression of FLAG-tagged PAX3-FOXO1 and (untagged) ETS1, followed by immunoprecipitation with FLAG antibody, co-immunoprecipitated ETS1 (Fig. 4C). Thus, ETS1 and PAX3-FOXO1 form a complex in a heterologous cell context, suggesting that they may interact directly. Taken together, our cistrome and biochemical studies indicate that ETS1 and PAX3-FOXO1 exist in a shared complex in FP-RMS, and that ETS1, potentially via direct protein-protein interactions, increases PAX3-FOXO1 levels on chromatin, including at enhancer elements controlling expression of important FP-RMS-promoting genes.

### YK-4-279 disrupts ETS1/PAX3-FOXO1 interactions, inhibits FP-RMS growth and invasive properties, and interferes with PAX3-FOXO1 gene regulation

The small molecule inhibitor YK-4-279 was originally identified as a compound able to inhibit critical interactions between EWS-FLI1, the driver oncofusion in Ewing Sarcoma, and RNA Helicase A [30]. FLI1 is a member of the ETS transcription family, and subsequent studies have shown broader activity of YK-4-279 against other ETS transcription factors in other cancers [31, 32]. Interestingly, recent studies demonstrated activity of YK-4-279 against critical ETS1/PAX3 interactions in melanoma [33]. We thus queried whether YK-4-279 could inhibit ETS1/PAX3-FOXO1 interactions, and oncogenic phenotypes, in FP-RMS.

In pilot growth assays, YK-4-279 inhibited the growth of Rh30 and Rh41 FP-RMS cells, with an IC_50_ of approximately 1.5 µM (MTT assay, 72 hours of drug treatment; Fig. S8A). Based on these data, we selected a drug concentration of 1 µM for our studies, as a dose within a relevant biological range in phenotypic studies. Treatment of Rh30 and Rh41 cells with 1 µM YK-4-279 did not impact total levels of ETS1 or PAX3-FOXO1 (or FOXO1; Fig. 4D). However, 1 µM YK-4-279 treatment disrupted ETS1/PAX3-FOXO1 interactions, as indicated by reduced co-immunoprecipitation of PAX3-FOXO1 (approximately 50% of DMSO control in Rh30 cells and 65% of DMSO control in Rh41 cells) using ETS1 antibody (Fig. 4D). Similar to the prior experiments (Fig. 4A), ETS1 also co-immunoprecipitated FOXO1, though less so in Rh30 cells, and this co-immunoprecipitation was less affected by 1 μM YK-4-279 (Fig. 4D). Furthermore, by CUT&RUN analysis, treatment of Rh30 and Rh41 cells with 1 µM YK-4-279 resulted in reduced levels of PAX3-FOXO1 at shared chromatin sites in both cell lines, again without impacting overall factor protein levels (Figs. 4E, S9 and S10). In Rh41 cells, this effect was accompanied by a more modest, concomitant, reduction in ETS1 chromatin levels (Fig. 4E and S11). Thus, treatment of FP-RMS cells with YK-4-279 interferes with ETS1/PAX3-FOXO1 interactions and displaces PAX3-FOXO1 from chromatin.

**Fig. 5:**
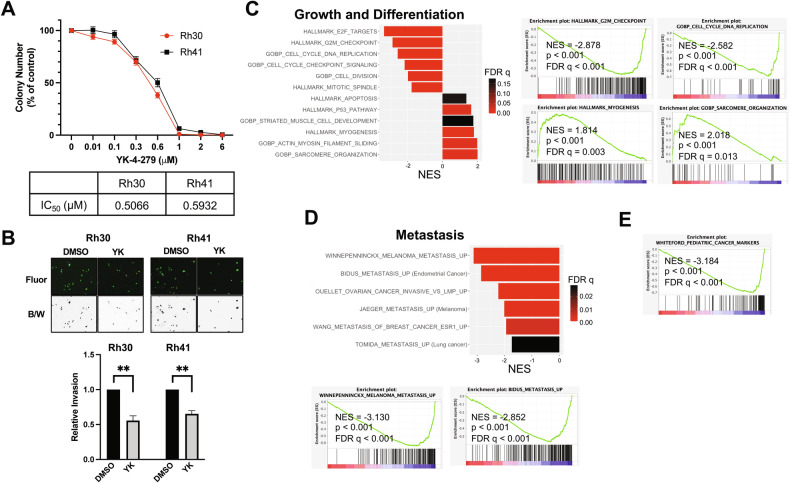
YK-4-279 inhibits FP-RMS growth and invasive properties, and related transcriptomes. **A** Effect of YK-4-279 treatment on FP-RMS growth in a colony formation assay. Rh30 and Rh41 cells were treated with the indicated doses of YK-4-279 every 3 days for 14 days. Data shown represent mean and s.e.m. of colony number, as percent of vehicle (DMSO) control, from three independent experiments, each performed in triplicate. IC_50_ values are shown below. **B** Effect of YK-4-279 treatment on FP-RMS transendothelial invasion. Rh30 and Rh41 cells were treated with 1 µM YK-4-279 or vehicle control for 8 hours, and then challenged to invade across a monolayer of HUVECs in the presence of 1 µM YK-4-279, or DMSO, for 16 hours. Top: Representative images of invaded cells, shown both as fluorescence images (Calcein AM dye-labeled cells; “Fluor”), and as pseudo-colored images (black on a white background; “B/W”), for greater ease of visualization. Bottom: Quantification of invasion data, plotted as mean and s.e.m. from three independent experiments, each performed in triplicate, with the control (DMSO) set to 1. **p < 0.01 (from two-tailed Welch’s t-test). **C**–**E** MSigDB gene sets related to growth and myogenic differentiation (C), metastasis (D), and pediatric cancer markers (E), in Rh30 cells following 1 µM YK-4-279 treatment for 24 hours, relative to vehicle control (DMSO), as determined by GSEA of RNAseq data. Summary plots, and selected individual GSEA plots, are shown. NES: Normalized Enrichment Score; p: nominal p-value; FDR q: False Discovery Rate (q-value).

We next further investigated YK-4-279 phenotypic effects in FP-RMS. We examined growth properties in Rh30 and Rh41 cells, using MTT assays of high-density growth and clonogenic (colony formation) assays of low-density growth. YK-4-279 inhibited FP-RMS cell growth properties in both assay types, with an average IC_50_ of approximately 1.5 µM in MTT assays (as already noted above; Fig. S8A) and 0.5 µM in clonogenic assays (Fig. 5A). We next asked whether YK-4-279 could inhibit motile/invasive properties, which underlie the aggressive metastatic biology of FP-RMS, and are promoted by both PAX3-FOXO1 [16] and ETS1 [14]. We selected 1 µM YK-4-279 for these studies as, at this concentration, the drug exerted negligible effects on Rh30 or Rh41 cell growth in the time frame of these assays (Fig. S8B). Strikingly, 1 µM YK-4-279 robustly inhibited transendothelial invasion (Fig. 5B), as well as cell migration and matrix invasion (Fig. S8C, D), in both cell lines. Thus, YK-4-279 inhibits growth and motile/invasive properties in FP-RMS, similar to the effects of PAX3-FOXO1 and ETS1 depletion.

To gain further insight into YK-4-279 action in FP-RMS, we examined the transcriptomic effects of drug treatment in Rh30 cells. Consistent with our phenotypic findings of growth inhibitory effects, transcriptome analysis revealed downregulation of gene sets related to cell cycle and replication upon YK-4-279 treatment (1 µM, as in phenotypic studies; Fig. 5C). Furthermore, as frequently observed in FP-RMS upon growth inhibition, as well as impairment of PAX3-FOXO1 action, YK-4-279 treatment also resulted in upregulation of gene sets related to myogenic differentiation (Fig. 5C). Strikingly, YK-4-279 treatment resulted in downregulation of gene sets related to cancer metastasis (Fig. 5D). This was concordant with our phenotypic findings of impaired motile/invasive properties in YK-4-279-treated cells (Fig. 5B), and was notably also observed in our prior studies of ETS1 knockdown in FP-RMS [14]. Also similar to our prior studies of ETS1 knockdown in FP-RMS, YK-4-279 treatment downregulated a group of genes commonly overexpressed in pediatric cancers relative to normal tissue [34] (Fig. 5E).

**Fig. 6:**
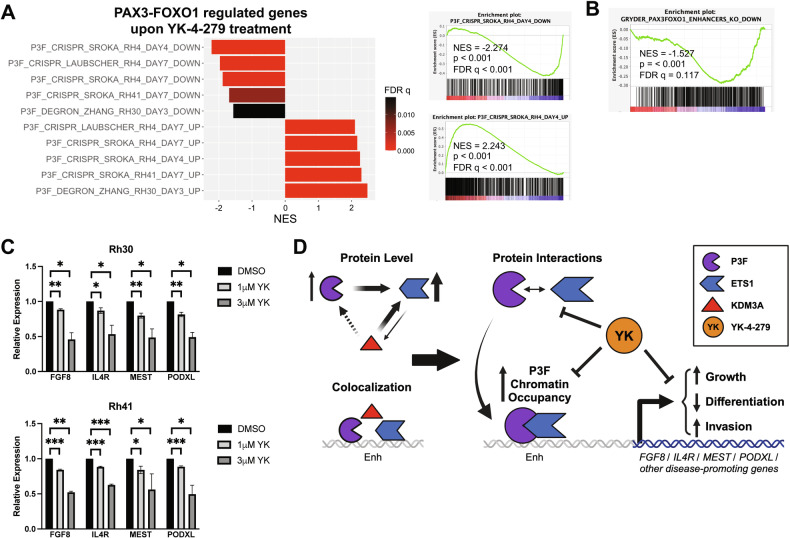
YK-4-279 interferes with PAX3-FOXO1 gene regulation. **A** GSEA analysis of RNAseq data from YK-4-279 treated Rh30 cells (1 µM, 24 hours; relative to DMSO control) against PAX3-FOXO1 regulated transcriptomes from three independent published studies (utilizing PAX3-FOXO1 sgRNA knockout in Rh4 and Rh41 cells [27, 35], and PAX3-FOXO1 degron-mediated depletion in Rh30 cells [36]). NES: Normalized Enrichment Score; p: nominal p-value; FDR: False Discovery Rate (q-value). Summary plot (left), and selected individual GSEA plots (right), are shown. **B** GSEA enrichment plot for same Rh30 YK-4-279 versus DMSO transcriptome against PAX3-FOXO1 enhancer-activated gene set [10]. **C** Relative expression of FGF8, IL4R, MEST, and PODXL upon YK-4-279 treatment (1 or 3 µM, each for 24 hours), compared to vehicle (DMSO) control, as determined by qRT-PCR, in Rh30 and Rh41 cells. Data shown represent mean and s.e.m. from three (1 µM drug versus DMSO), or two (3 µM drug versus DMSO) independent experiments, each performed in triplicate, with control (DMSO) set to 1. RPL19 (confirmed to be invariant upon YK-4-279 treatment in RNAseq analysis) was used as an internal control. *p < 0.05; **p < 0.01; ***p < 0.001; from two-tailed Welch’s t-test, comparing each treatment (1 µM or 3 µM drug) with its own vehicle (DMSO) control). **D** Schematic diagram of proposed mechanisms based on our findings. KDM3A and PAX3-FOXO1 (P3F) each upregulate ETS1 expression (our previous findings [14, 15]). Herein, we further show that ETS1 also upregulates KDM3A and, in some FP-RMS cell contexts, KDM3A additionally upregulates PAX3-FOXO1. KDM3A and ETS1 co-localize with PAX3-FOXO1 at regulatory elements, including enhancers (Enh), of important FP-RMS growth and invasion/metastasis-promoting genes like FGF8, IL4R, MEST, and PODXL. Through protein-protein interactions, ETS1 also augments PAX3-FOXO1 chromatin occupancy. Together, these mechanisms serve to amplify PAX3-FOXO1 action, resulting in increased expression of genes controlling growth and invasion/metastasis (including FGF8, IL4R, MEST, and PODXL), as well as inhibiting differentiation, thereby promoting FP-RMS progression. Through interference with ETS1/PAX3-FOXO1 interactions, YK-4-279 displaces PAX3-FOXO1 from chromatin, inhibits PAX3-FOXO1 mediated gene regulation, impairs growth and invasive properties, and promotes differentiation, thereby reducing FP-RMS progression.

Thus, YK-4-279 treatment downregulates cancer-relevant genes related to cell proliferation and metastasis, and upregulates genes related to myogenic differentiation, which represent key oncogenic parameters related to FP-RMS pathogenesis and progression. To determine whether YK-4-279 specifically interferes with PAX3-FOXO1 action, as suggested by our cistrome and biochemical studies, we examined the effects of drug treatment on PAX3-FOXO1-dependent gene expression. To do this, we queried PAX3-FOXO1-dependent transcriptomes as defined in three recent, independent, studies, utilizing PAX3-FOXO1 sgRNA knockout in Rh4 and Rh41 cells [27, 35], and PAX3-FOXO1 degron-mediated depletion in Rh30 cells [36]. Strikingly, YK-4-279 treatment resulted in downregulation of PAX3-FOXO1-activated genes, and upregulation of PAX3-FOXO1-repressed genes, in data from all three studies (Fig. 6A), consistent with inhibition of PAX3-FOXO1-dependent gene regulation. Furthermore, similar to ETS1 knockdown (Fig. 1C), YK-4-279 treatment resulted in downregulation of PAX3-FOXO1 enhancer-activated genes (Fig. 6B). Lastly, and in further support of on-target, disease-relevant effects, YK-4-279 treatment resulted in dose-dependent downregulation of FGF8, IL4R, MEST, and PODXL gene expression in both Rh30 and Rh41 cells (Fig. 6C). Taken together, our data indicate that YK-4-279 disrupts ETS1/PAX3-FOXO1 interactions, inhibits FP-RMS growth and invasive properties, and interferes with disease-relevant PAX3-FOXO1 gene regulation.

## Discussion

PAX3-FOXO1-driven fusion-positive RMS (FP-RMS) is an aggressive disease with strong tendency toward metastasis, progression, and poor patient outcomes. As revealed by recent studies, PAX3-FOXO1-driven FP-RMS fundamentally relies on epigenetic mechanisms for pathogenesis [10]. Details of these mechanisms, which may pave the way toward new treatment paradigms, are gradually emerging. However, much remains to be learned. Important areas of investigation toward this end include: how PAX3-FOXO1 interacts with other chromatin and transcription factors to drive disease pathogenesis; how such mechanisms promote aggressive biological properties leading to disease progression.

Our previous studies identified the chromatin factor KDM3A and the ETS1 transcription factor as part of a novel epigenetic axis that functionally and transcriptomically interfaces with PAX3-FOXO1 to promote growth and metastatic properties in FP-RMS [14, 15]. In this study, we used cistrome profiling to demonstrate that this functional and transcriptomic interface involves direct mechanisms, namely co-association with shared regulatory elements, including enhancers. Furthermore, we show that ETS1, through apparent protein-protein interactions, augments PAX3-FOXO1 chromatin occupancy. We have previously shown that PAX3-FOXO1 upregulates ETS1 expression in FP-RMS [14]. Thus, ETS1 is induced by PAX3-FOXO1, and, in turn, increases PAX3-FOXO1 levels on chromatin. In this manner, ETS1 effectively acts as PAX3-FOXO1-induced “amplifier” of PAX3-FOXO1 action in FP-RMS. This amplification mechanism is further reinforced by KDM3A, which also upregulates ETS1 expression in FP-RMS [15] (and Fig. S7).

We demonstrate that ETS1 and PAX3-FOXO1 interact and exist in a shared complex in FP-RMS, and that this interaction can be disrupted by YK-4-279, a small molecule inhibitor of ETS protein-protein interactions. In functional and transcriptomic studies, we show that YK-4-279 robustly inhibits growth and motile/invasive properties, as well as promotes myogenic differentiation, in FP-RMS. Concurrent with these effects, and at a biologically relevant dose, YK-4-279 also opposes PAX3-FOXO1-dependent transcriptional regulation. Our studies thus identify YK-4-279 as a novel PAX3-FOXO1 and FP-RMS pharmacologic inhibitor. Given that YK-4-279 is a clinically relevant compound (with TK-216, its clinical analog, in phase II clinical trials for Ewing Sarcoma [37]), further evaluation of its activity in FP-RMS is warranted.

Further studies of ETS1/PAX3-FOXO1 interactions, and their susceptibility to YK-4-279 inhibition, can be expected to inform mechanisms of PAX3-FOXO1 chromatin complex assembly and maintenance, and ways that such mechanisms could be targeted in FP-RMS. In other (non-RMS) biological contexts, ETS1 is able to interact with both (wild-type, full length) PAX3 [38] and FOXO1 [28, 29]. Thus, both the PAX3 and FOXO1 components of the PAX3-FOXO1 fusion protein could potentially contribute to the ETS1 interaction. Our studies show that ETS1 is required to maintain high levels of PAX3-FOXO1 on chromatin. This raises the question of whether ETS1 is also needed for initial PAX3-FOXO1 recruitment to chromatin during disease initiation, a topic of interest in future investigations. It will also be important to further define how YK-4-279 disrupts P3F chromatin regulatory complexes in FP-RMS. We show that YK-4-279 is able to displace PAX3-FOXO1 from chromatin in both Rh41 and Rh30 cells. Importantly, the loss of PAX3-FOXO1 from chromatin is concordant with the observed phenotypic and gene regulatory effects in our studies. Interestingly, at the same drug dose, YK-4-279 displaces ETS1 from chromatin in Rh41, but not Rh30, cells. These findings are not necessarily surprising since YK-4-279, a protein-protein interaction inhibitor, could in principle interfere with chromatin complex function with, or without, the loss of any one individual complex component from chromatin. The differential effects on ETS1 in the two cell lines could be due to precise composition and levels of chromatin complex components, post-translational modifications, complex dynamics, 3D chromatin configuration, or other factors, which may not be identical in different patient-derived cell lines. Further studies to understand the precise effects of YK-4-279 on P3F chromatin complexes in FP-RMS can be expected to shed important light on mechanisms of drug action.

Our studies provide evidence for a YK-4-279 “on-target” mechanism of action involving disruption of ETS1/PAX3-FOXO1 interactions. YK-4-279 of course has the potential to disrupt other ETS protein-protein interactions, including in FP-RMS. As such, we cannot exclude a potential role for other ETS factors, and other ETS protein-protein interactions, in the biological effects of this drug in FP-RMS. Notably, in the context of a drug with an already-defined safety profile in humans, broader-scope activity in the tumor could be an additional benefit, rather than a liability. That is, demonstration of additional efficacious mechanisms in the tumor should not raise additional safety concerns outside the tumor, since the latter are already known (from completed Phase I clinical trial studies).

Members of the Jumonji-domain histone demethylase (JHDM) family have been of interest in pediatric cancer, including RMS [39]. This includes members of the KDM3 and KDM4 families, which share activity against the H3K9 histone methyl mark (H3K9me1/2 for KDM3s, and H3K9me2/3 for KDM4s). Our studies identified a role for KDM3A in Ewing Sarcoma [13], and subsequently RMS [14, 15], and demonstrated efficacy of the pan-JHDM inhibitor JIB-04 [40], in both cancer types [15, 41]. A subsequent study demonstrated a role for KDM4B, and KDM4 inhibition using the small molecule inhibitor QC6352, in FP-RMS [42]. Most recently, another study demonstrated a role for KDM3B, and inhibition using the small molecule inhibitor P3FI-63 (and the optimized compound P3FI-90), in FP-RMS [43]. Interestingly, P3FI-63 also inhibits KDM3A and shares transcriptomic effects with JIB-04 [43]. Targeting of the KDM3/4 families thus remains of interest in FP-RMS, and the above studies provide multiple alternatives.

Our mechanistic studies of KDM3A imply more complex modes of action in FP-RMS that will require further study. We show that in some FP-RMS, KDM3A upregulates P3F levels, and consequent P3F chromatin occupancy. This provides another mechanism that could potentially be exploited to inhibit P3F action in some FP-RMS. On the other hand, our studies suggest that, in some contexts, KDM3A may be able to co-regulate FP-RMS genes with P3F by mechanisms other than control of P3F levels or chromatin occupancy. Our studies also reveal interesting differences between the effects of KDM3A depletion (and accompanying ETS1 downregulation), and ETS1 depletion (and accompanying KDM3A downregulation), upon P3F chromatin occupancy. Some of these differences could be due to the depth of KDM3A and ETS1 level reduction, which are not the same in the respective experimental manipulations. It is also possible that the sequence of factor level reduction is important, and results in non-identical chromatin effects. Lastly, our studies also suggest possible regulation of KDM3A chromatin occupancy by ETS1. Such regulation could be relevant not only in FP-RMS, but also to KDM3A/ETS1 disease-promoting biology in FN-RMS [15, 44]. These and other mechanisms merit further investigation to understand the disease-promoting effects of KDM3A in RMS.

The vast majority of studies of PAX3-FOXO1 and related epigenetic mechanisms in FP-RMS have focused on growth and differentiation phenotypes. Such mechanisms clearly play important roles in FP-RMS pathogenesis. However, given the strong propensity of FP-RMS toward dissemination and metastasis, motile/invasive mechanisms are undoubtedly critical as well, especially in disease progression. It is thus notable that KDM3A and ETS1, which themselves promote invasive and metastatic properties in FP-RMS [14], coregulate, along with PAX3-FOXO1, important, known, pro-invasive/metastatic genes specifically upregulated in FP-RMS, including FGF8, IL4R, and MEST. Our functional studies herein indicate that the transmembrane protein PODXL also belongs to this group of FP-RMS-upregulated, pro-invasive and pro-metastatic genes. Notably, PODXL promotes FP-RMS transendothelial invasion, a key biological property in sarcoma dissemination, and is a cell surface-expressed protein. PODXL thus presents a new candidate therapeutic target in FP-RMS, for inhibition of metastatic properties. Together, genes such as FGF8, IL4R, MEST and PODXL, and their associated pathways, may importantly contribute to the basis of the aggressive biological properties of FP-RMS. Notably, the same genes, as well as FP-RMS motile and invasive properties, are vulnerable to YK-4-279 inhibition, further supporting rationale for more studies of this drug in this disease. On the other hand, our studies indicate that BRG1, another epigenetic regulator recently shown to interact with PAX3-FOXO1, and to promote growth, and inhibit differentiation, in FP-RMS [26, 27], does not regulate motile or invasive properties in FP-RMS. Interestingly, and concordant with these findings, BRG1 appears to exhibit more complex regulation of FP-RMS pro-invasive/metastatic genes such as FGF8, IL4R, MEST, and PODXL, relative to PAX3-FOXO1, KDM3A and ETS1 (Fig. S6F).

In summary, we define new mechanisms by which KDM3A and ETS1 interface with the PAX3-FOXO1 disease-driving oncofusion to promote FP-RMS. We show that ETS1, a factor induced by PAX3-FOXO1 and KDM3A, complexes with PAX3-FOXO1 and increases its chromatin occupancy, thus effectively functioning as an amplifier of PAX3-FOXO1 action in FP-RMS. Furthermore, we identify the clinically relevant small molecule YK-4-279, an ETS protein-protein interaction disruptor, as a novel inhibitor of ETS1/PAX3-FOXO1 interactions, PAX3-FOXO1 chromatin localization and gene regulation, and FP-RMS growth and invasive properties. Together, these findings advance understanding of PAX3-FOXO1-driven FP-RMS and identify new therapeutic options for this aggressive disease.

## Materials and methods

Materials and Methods can be found in Supplementary Materials.

## Supplementary information

Supplementary Materials

## Data Availability

Transcriptome and cistrome data have been deposited in the NCBI Gene Expression Omnibus database (accession numbers GSE279335 and GSE279336; superseries GSE279337).
