# Supplementary material for: Dependence of PAX3-FOXO1 chromatin occupancy on ETS1 at important disease-promoting genes exposes new targetable vulnerability in Fusion-Positive Rhabdomyosarcoma
Source: Oncogene. 2024 Oct 24;44(1):19–29. doi: 10.1038/s41388-024-03201-2 (PMC11700839; doi:10.1038/s41388-024-03201-2)
Supplement: Supplementary file 1 — Supplementary Materials [file 41388_2024_3201_MOESM1_ESM.pdf]

Supplementary Materials

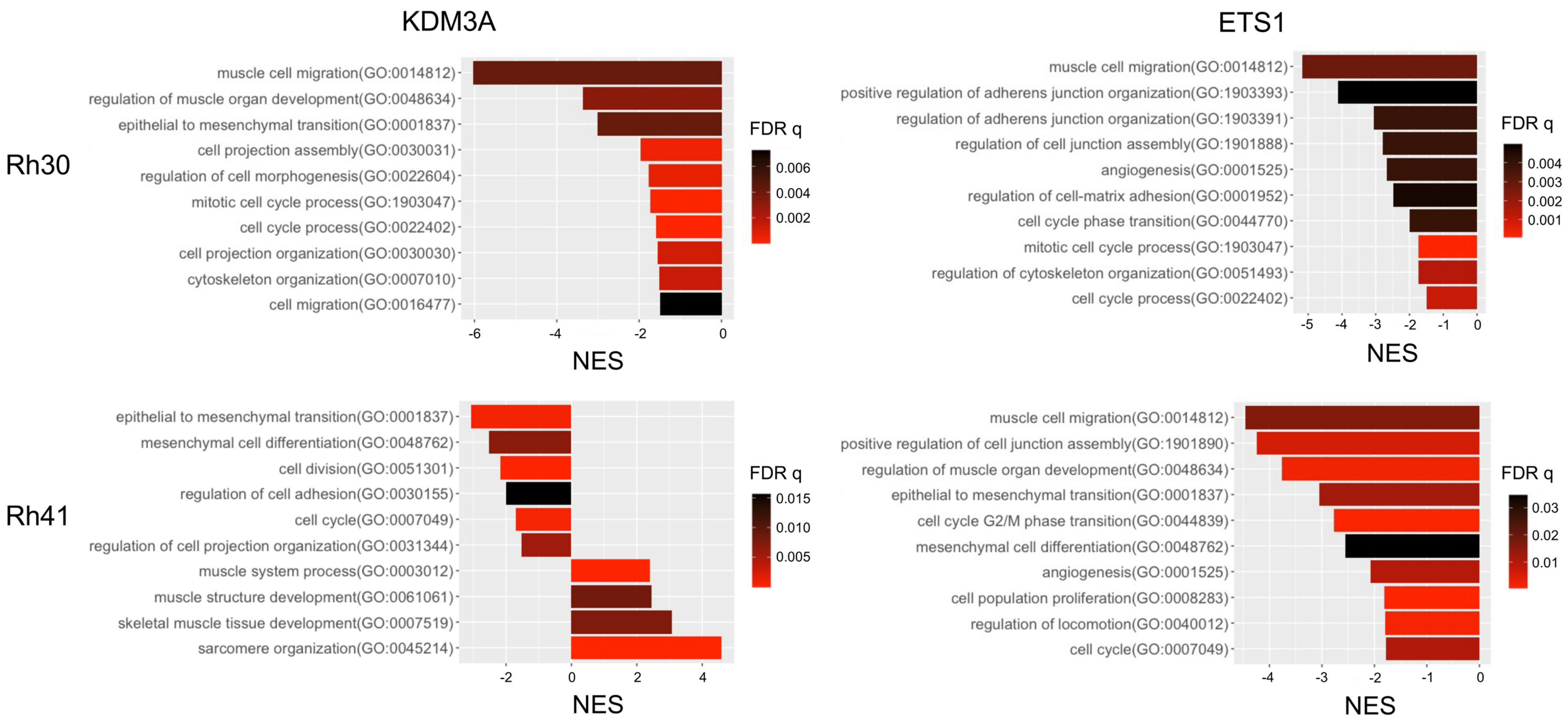

Supplementary Figure S1

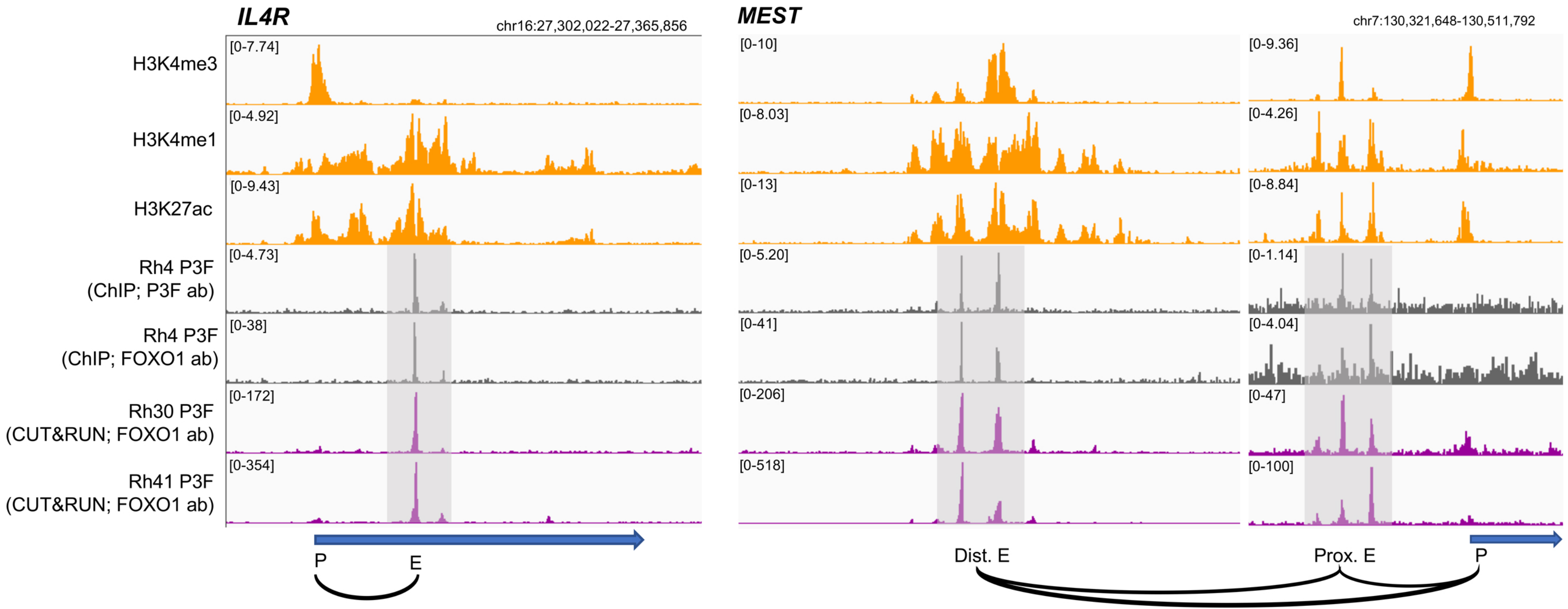

Supplementary Figure S2

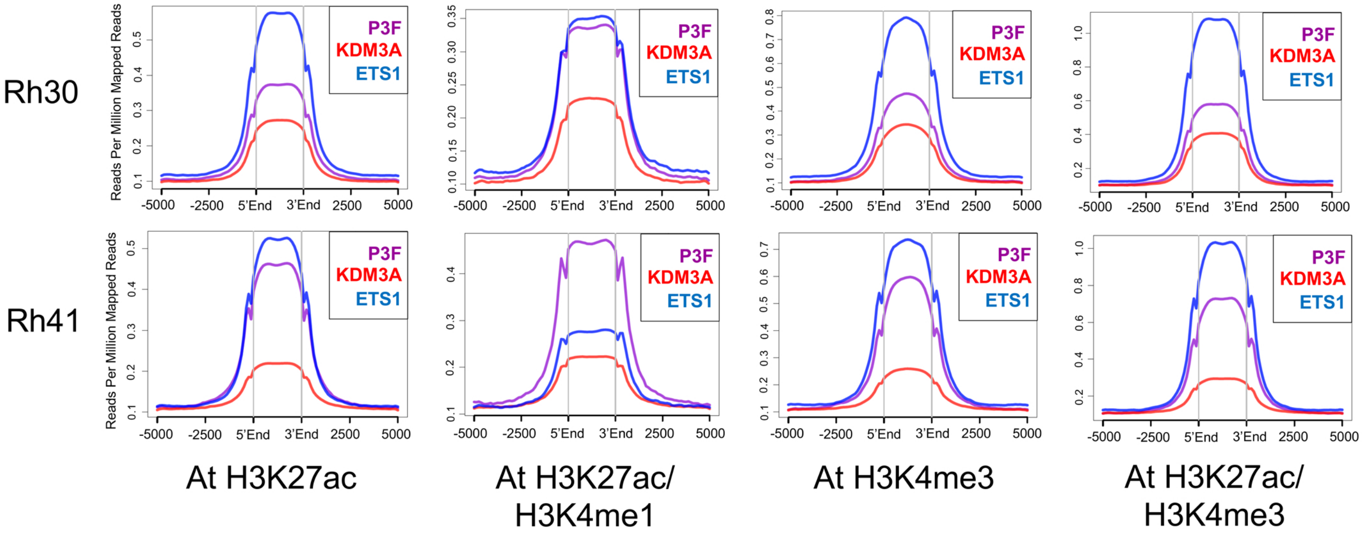

Supplementary Figure S3

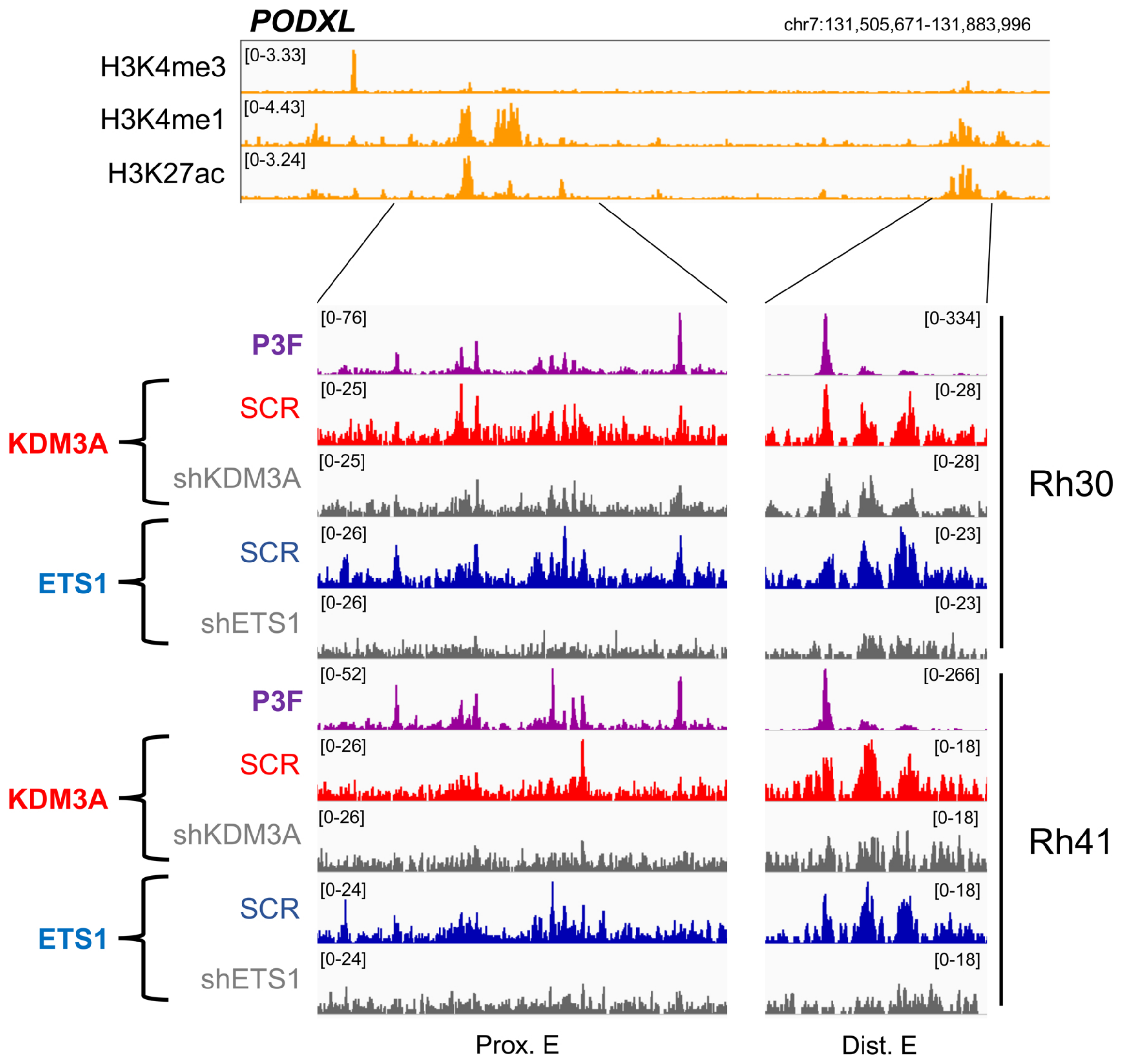

Supplementary Figure S4

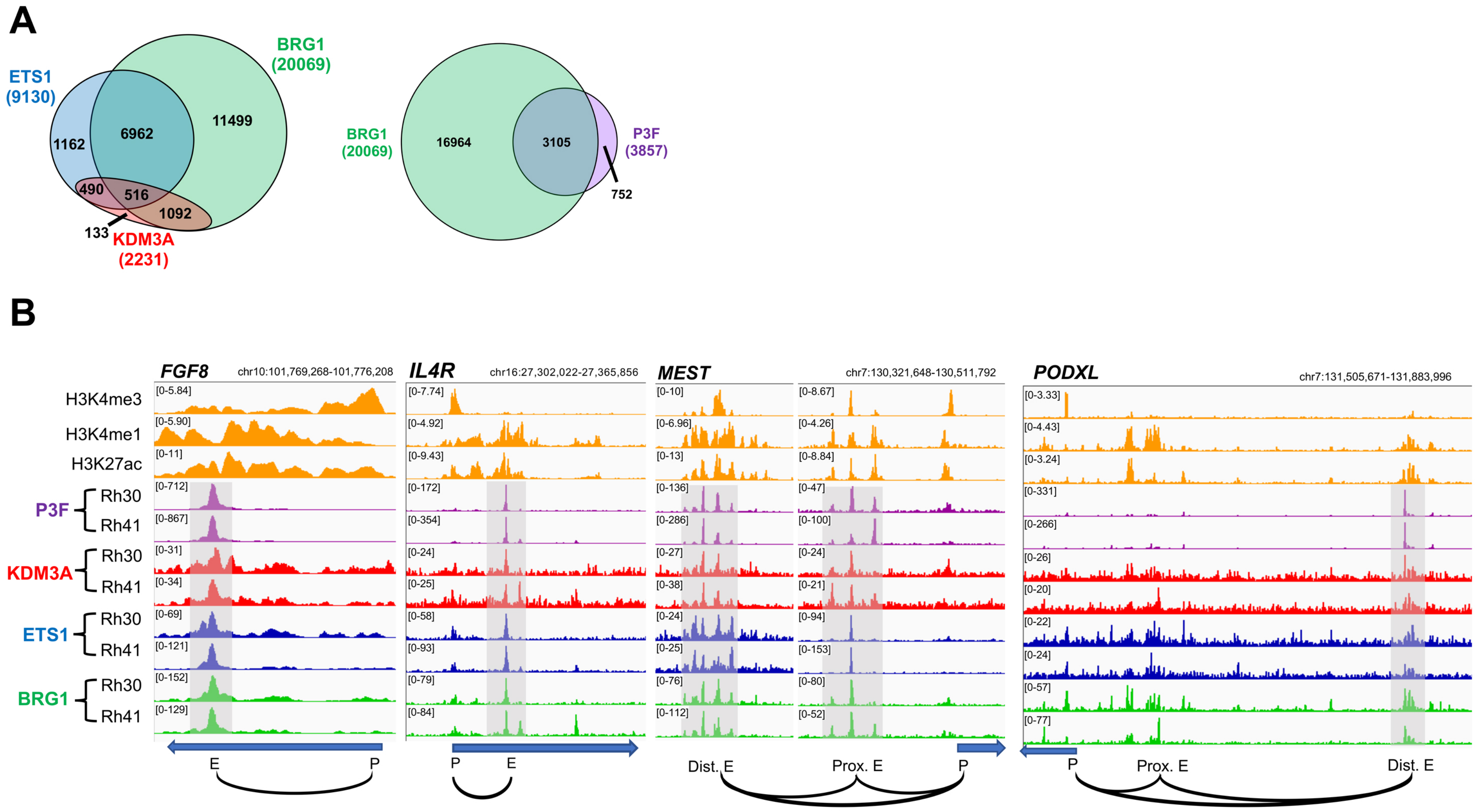

Supplementary Figure S5

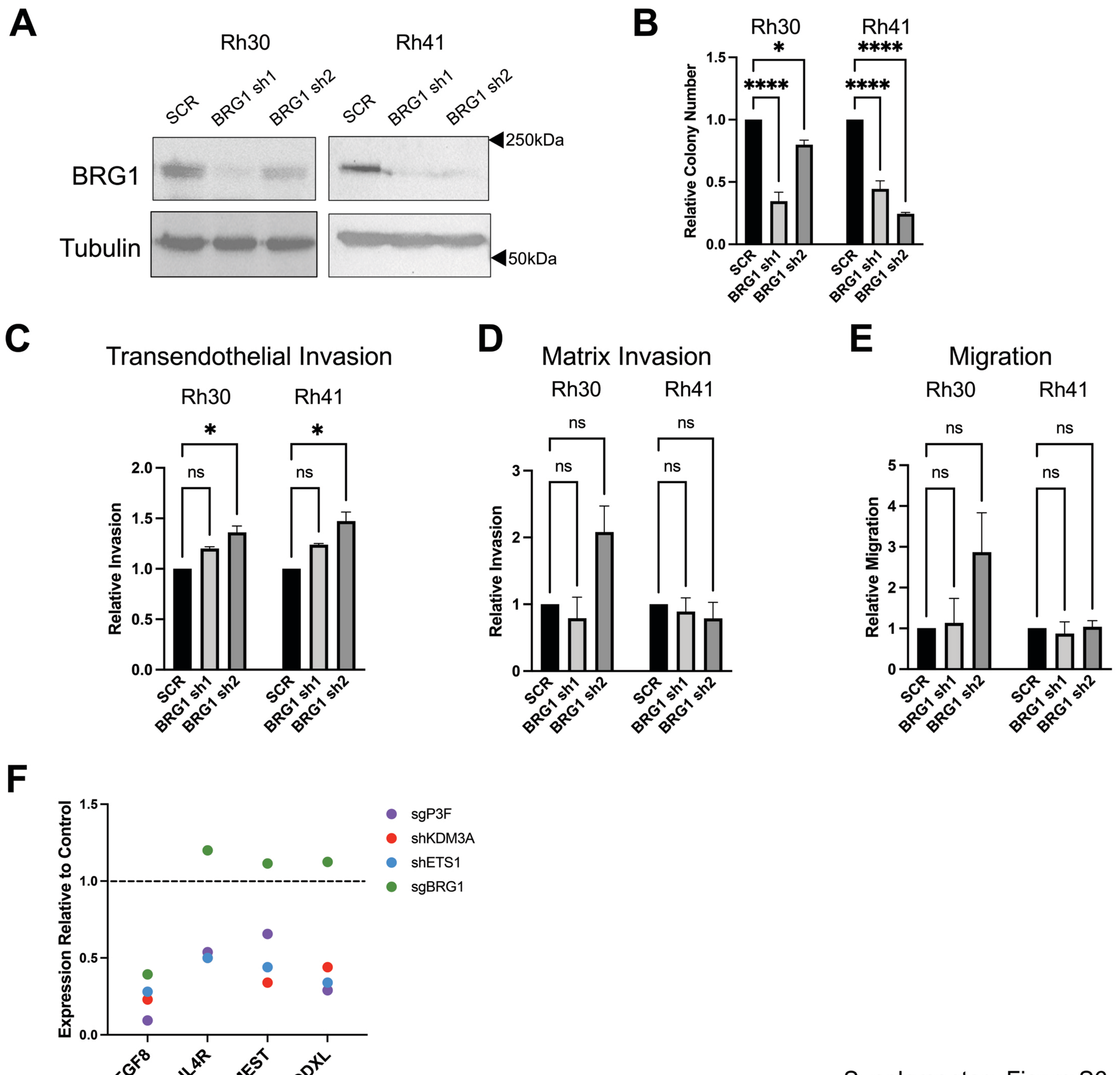

Supplementary Figure S6

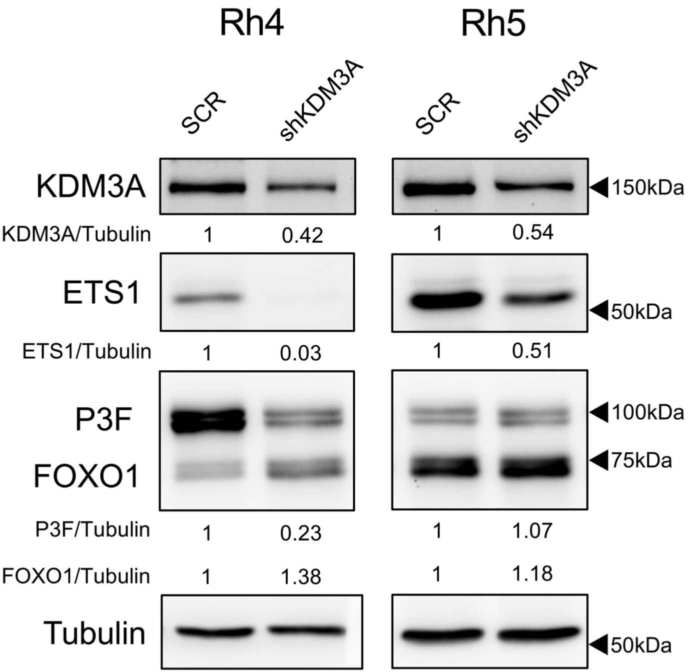

Supplementary Figure S7

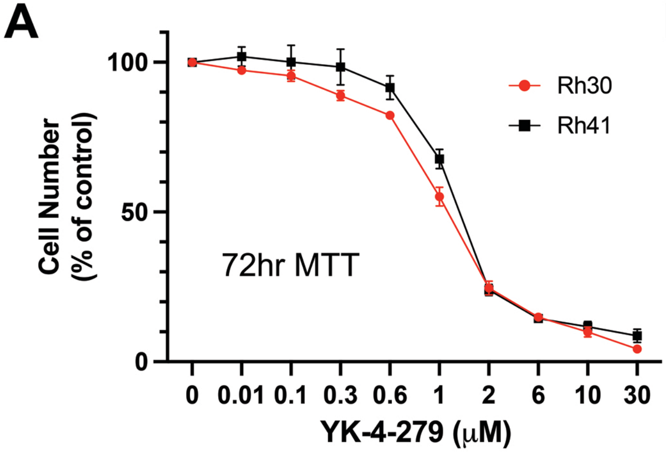

	Rh30	Rh41
IC_{50} (μM)	1.293	1.673

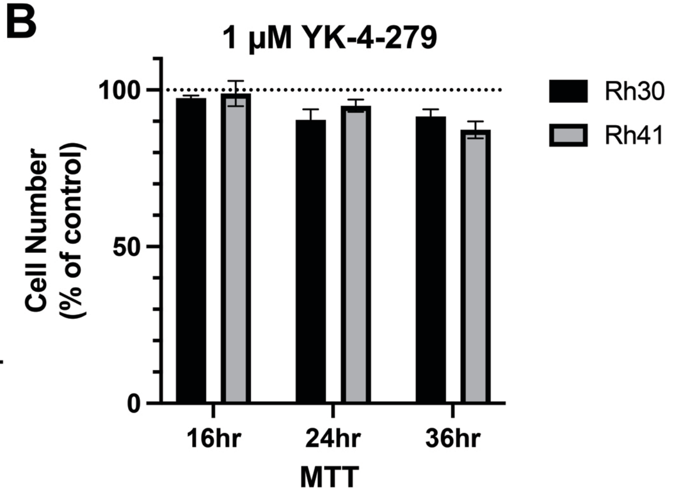

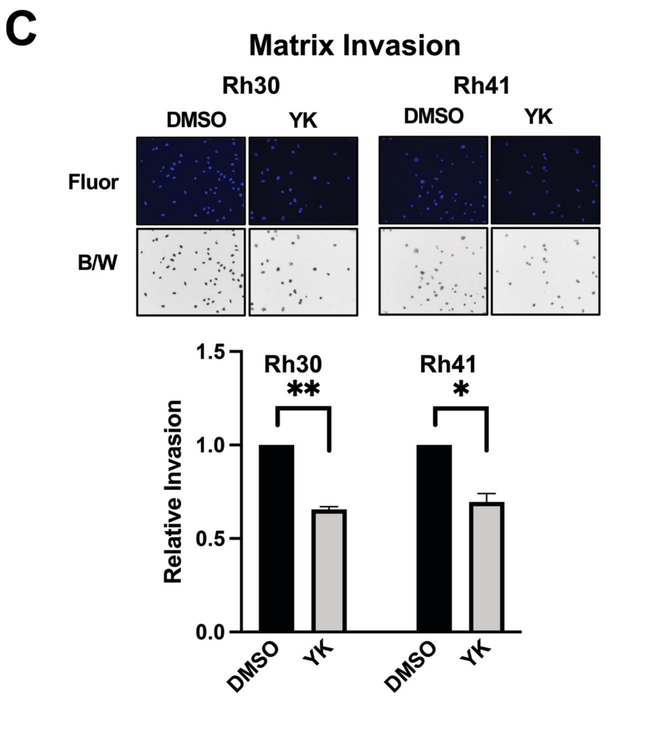

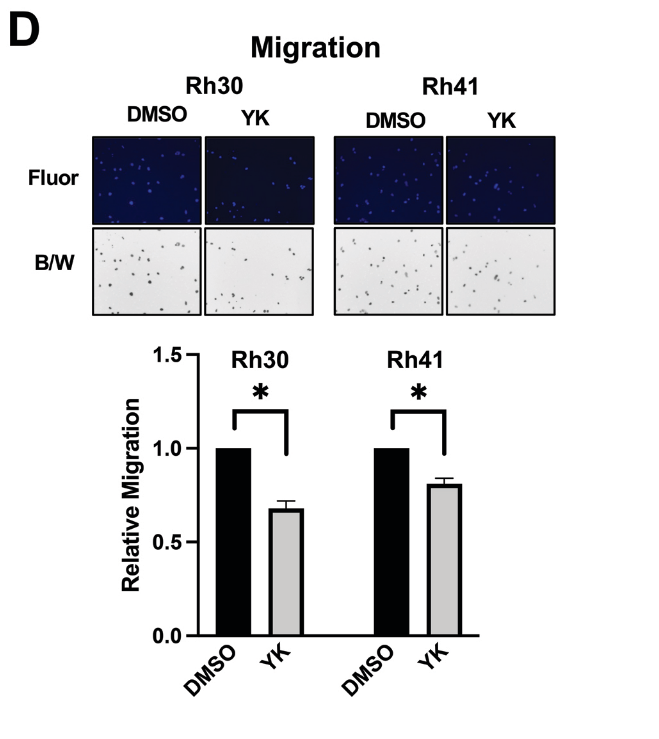

Supplementary Figure S8

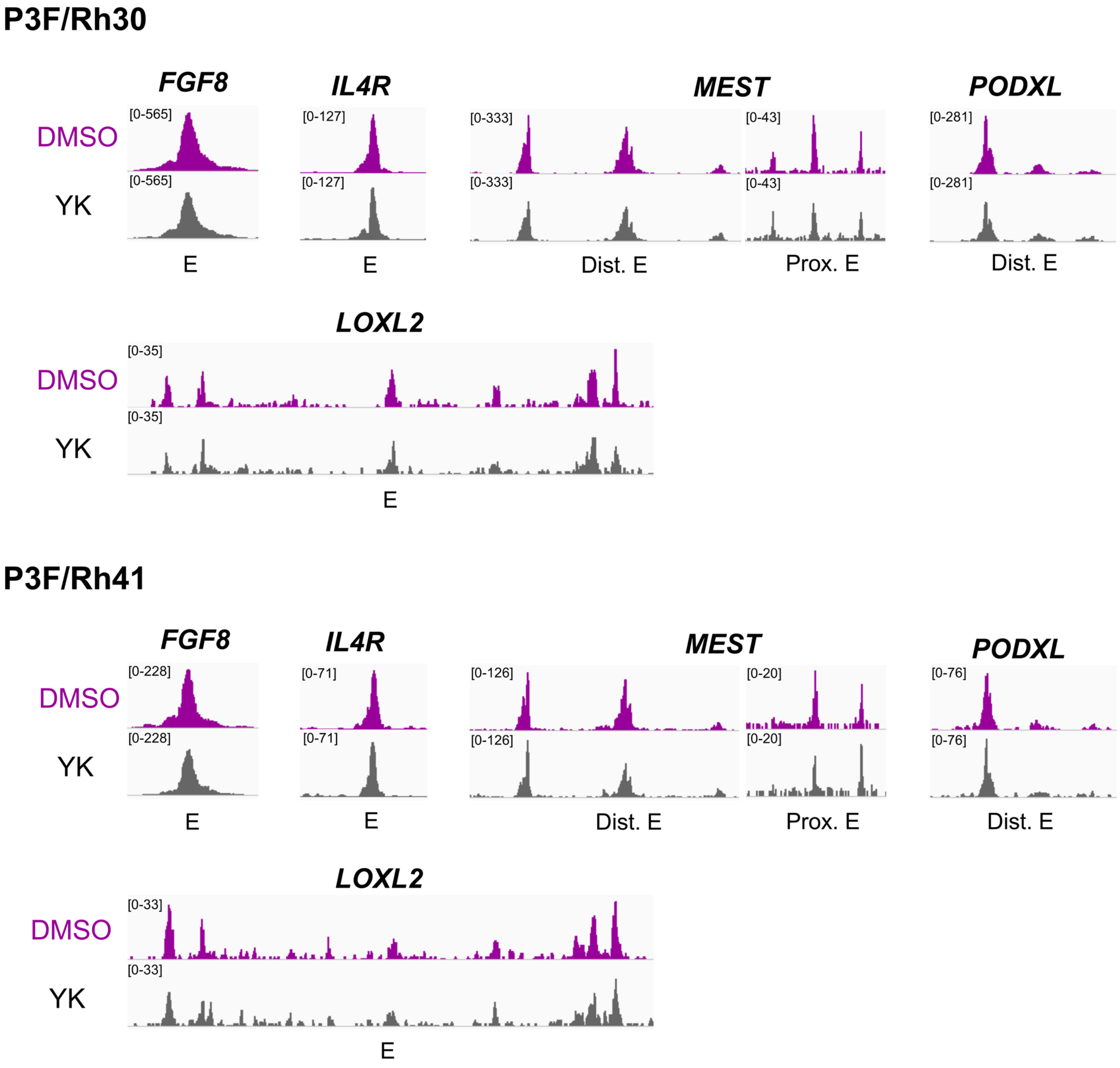

Supplementary Figure S9

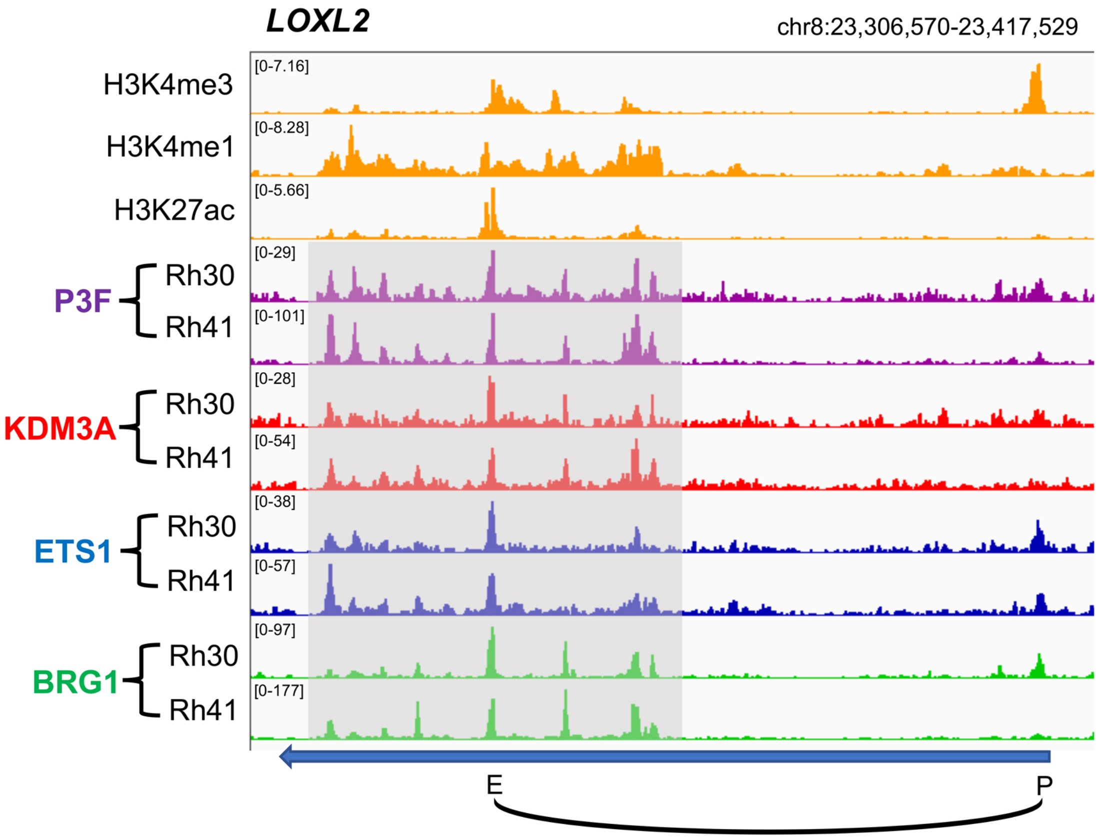

Supplementary Figure S10

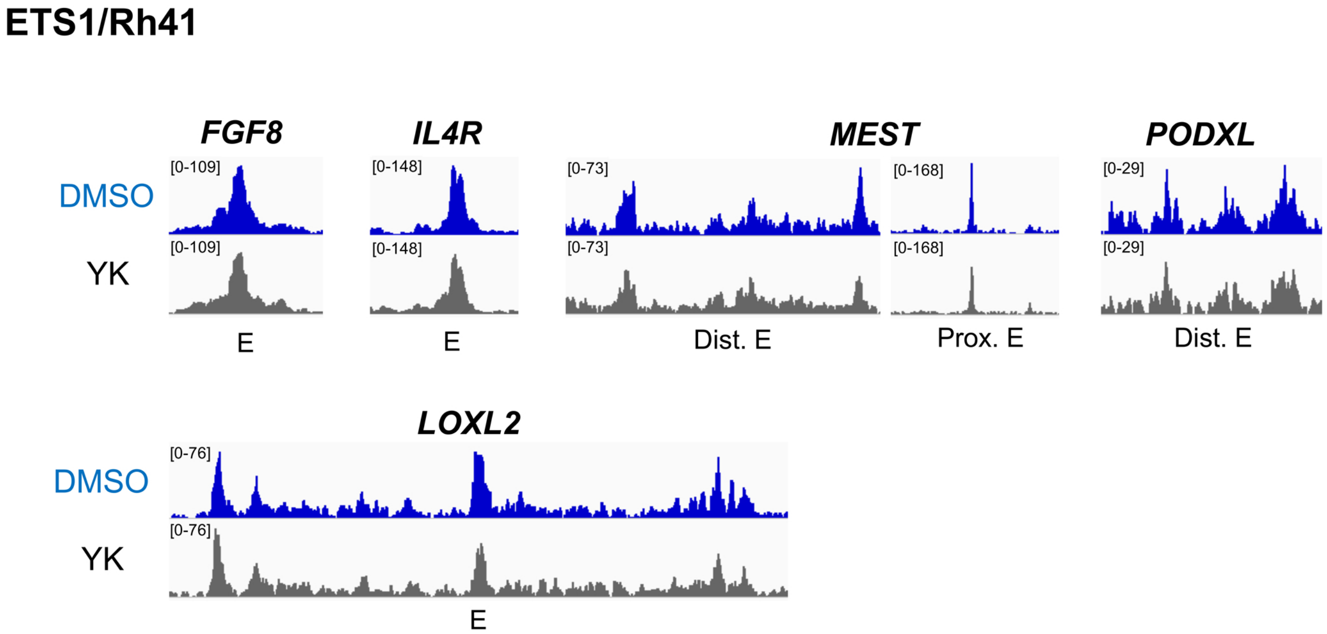

Supplementary Figure S11

Supplementary Figure Legends

Supplementary Figure S1: Integrative cistromic and transcriptomic analysis of KDM3A and ETS1 in FP-RMS reveals direct regulatory control of cancer-promoting pathways. Rh30 and Rh41 FP-RMS cell line, KDM3A and ETS1, CUT&RUN cistrome data integrated with our prior transcriptome data from the same cell lines [1], using Cistrome-GO [2]. NES: Normalized Enrichment Score; FDR q: False Discovery Rate (q-value).

Supplementary Figure S2: PAX3-FOXO1 cistromes generated using CUT&RUN and FOXO1 antibody are congruent with published PAX3-FOXO1 cistromes from FP-RMS cells. Comparison of PAX3-FOXO1 (P3F) cistrome data from: Rh4 cells, generated by ChIP-seq using P3F junction antibody [3]; Rh4 cells, generated by ChIP-seq using C-terminal FOXO1 antibody [4]; Rh30 and Rh41 cells, generated by CUT&RUN and the same C-terminal FOXO1 antibody (our data herein). Reference FP-RMS histone mark tracks (H3K4me3, H3K4me1, and H3K27ac) are from [5], via CistromeDB. Blue arrows depict transcript direction. Black curved lines denote chromatin regional associations (Rh30 Hi-C data from ENCODE3, generated by Dekker Laboratory). “P”: promoter; “E”: enhancer; “Prox.”: proximal; “Dist.”: distal.

Supplementary Figure S3: Genomic distribution of PAX3-FOXO1, KDM3A, and ETS1 at FP-RMS H3K27ac, H3K27ac/H3K4me1, H3K4me3, and H3K27ac/H3K4me3 regulatory elements. FP-RMS histone mark data from [5], via CistromeDB; histone mark overlap analysis performed using BedSect [6]. Plots generated using ngs.plot analysis of our CUT&RUN data.

Supplementary Figure S4: PAX3-FOXO1, KDM3A (from control (SCR) and knockdown (shKDM3A) cells; note partial knockdown in both cell lines (Figure 3A)), and ETS1 (from control (SCR) and knockdown (shETS1) cells) cistrome tracks, along with reference FP-RMS histone mark tracks (H3K4me3, H3K4me1, and H3K27ac; from [5], via CistromeDB) at the PODXL proximal and distal enhancer loci (as shown in Fig. 2A), in Rh30 and Rh41 FP-RMS cells, as determined by CUT&RUN cistrome profiling (with internal normalization for KDM3A and ETS1). "E": enhancer; "Prox.": proximal; "Dist.": distal.

Supplementary Figure S5: BRG1 shows broad genomic distribution in FP-RMS, including majority of KDM3A, ETS1, and PAX3-FOXO1 bound loci. Peak overlap analysis (A) and representative cistrome track visualization (B), as in Figure 1.

Supplementary Figure S6: BRG1 does not promote invasive properties in FP-RMS. (A) BRG1 shRNA-mediated knockdown in Rh30 and Rh41 cells, as determined by immunoblotting, with tubulin as loading control; SCR: Scrambled shRNA negative control. (B) Colony formation assay data, performed and analyzed as in Figure 2. Mean and s.e.m. of three independent experiments, each performed in triplicate, with control set to 1. *$p < 0.05$; ****$p < 0.0001$ (from one-way ANOVA with multiple comparisons). (C-E) Transendothelial invasion (C), matrix invasion (D), and migration (E) assay data, each representing mean and s.e.m. from three independent experiments, each performed in triplicate, with control (SCR) set to 1. "ns": not significant ($p>0.05$); *$p < 0.05$ (from one-way ANOVA with multiple comparisons). (F) Mean fractional expression of the indicated genes (FGF8, IL4R, MEST, and PODXL) following factor knockdown (KDM3A and ETS1 [1]), or knockout (PAX3-FOXO1 (P3F) and BRG1 [7]), relative to control (RNAseq data).

Supplementary Figure S7: Effects of KDM3A knockdown on KDM3A, ETS1, PAX3-FOXO1, and FOXO1 total protein levels, as determined by immunoblotting and quantified by densitometry (Factor/Tubulin), in FP-RMS Rh4 and Rh5 cells.

Supplementary Figure S8: YK-4-279 inhibits FP-RMS growth and invasive properties. (A) Effect of YK-4-279 treatment on FP-RMS growth in MTT assay. Rh30 and Rh41 cells were treated with the indicated doses of YK-4-279 one day following plating, cultured for an additional 72 hours, and then subjected to MTT assay. Data shown represent mean and s.e.m. of MTT signal, as percent of vehicle control (DMSO), from three independent experiments, each performed in five replicates. IC_{50} values are shown below. (B) Treatment with 1 μM YK-4-279 exerts minimal effects on Rh30 and Rh41 cell number at 16, 24, and 36 hours, as determined by MTT assay, relative to vehicle (DMSO) control. Data shown represent mean and standard deviation from five replicates each. These data are in reference to migration and invasion assays in Figure 5B, and panels C and D below (migration assay = 20 hours total drug treatment; transendothelial invasion assay = 24 hours total drug treatment; matrix invasion assay = 32 hours total drug treatment). (C) Matrigel invasion assay. Rh30 and Rh41 cells were pre-treated with 1 μM YK-4-279 or vehicle control (DMSO) for 8 hours, then challenged to invade across Matrigel matrix in a transwell in the presence of 1 μM YK-4-279, or DMSO, for 24 hours. Top: Representative images of invaded cells, shown both as fluorescence images (DAPI-labeled nuclei; “Fluor”), and as pseudo-colored images (black on a white background; “B/W”), for greater ease of visualization. Bottom: Quantification of invasion data, plotted as mean and s.e.m. from two independent experiments, each performed in triplicate, with control (DMSO) set to 1. *p < 0.05; **p < 0.01 (from two-tailed Welch’s t-test). (D) Transwell migration assay. Rh30 and Rh41 cells were pre-treated with 1 μM YK-4-279 or vehicle control (DMSO) for 8 hours, then challenged to

invade across a transwell membrane in the presence of 1 μM YK-4-279, or DMSO, for 12 hours. Top: Representative images, presented as in “C”. Bottom: Quantification of migration data, plotted as mean and s.e.m. from two independent experiments, each performed in triplicate, with control set to 1. *$p < 0.05$ (from two-tailed Welch’s t-test).

Supplementary Figure S9: (A) CUT&RUN data for PAX3-FOXO1 from DMSO control and YK-4-279 treated Rh30 and Rh41 FP-RMS cells (same data as in Figure 4E), visualized at enhancer regulatory elements of the FGF8, IL4R, MEST, PODXL, and LOXL2 genes, using IGV. (LOXL2 is an additional PAX3-FOXO1, KDM3A and ETS1 co-bound (Fig. S9) and co-regulated [1], known metastasis-promoting [8], gene in FP-RMS.) “E”: enhancer; “Prox.”: proximal; “Dist.”: distal.

Supplementary Figure S10: PAX3-FOXO1, KDM3A, ETS1, and BRG1 CUT&RUN cistrome tracks at the LOXL2 gene locus in FP-RMS Rh30 and Rh41 cells, plotted and visualized as in Figure 1E. “P”: promoter; “E”: enhancer.

Supplementary Figure S11: CUT&RUN data for ETS1 from DMSO control and YK-4-279 treated Rh41 FP-RMS cells (same data as in Figure 4E), visualized at enhancer regulatory elements of the FGF8, IL4R, MEST, PODXL, and LOXL2 genes, using IGV. “E”: enhancer; “Prox.”: proximal; “Dist.”: distal.

Supplementary Table S1

Primers used (sequence 5' -> 3')

	Forward	Reverse
**FGF8**	GACCCCTTCGCAAAGCTCAT	CCGTTGCTCTTGGCGATCA
**IL4R**	CGTGGTCAGTGCGGATAACTA	TGGTGTGAACTGTCAGGTTTC
**MEST**	ATCGGGTGATTGCCCTTGATT	GAAAGAAGGTTGATCCTGCGG
**PODXL**	TCCCAGAATGCAACCCAGAC	GGTGAGTCACTGGATACACCAA
**RPL19**	GCCCATCTTTGATGAGCTTC	GTGGCAAGAAGAAGGTCTGG

Materials and Methods

Cell lines and drugs

The patient-derived FP-RMS cell lines Rh30 and Rh41 have been described previously [9]. Rh4 and Rh5 FP-RMS cell lines were kindly provided by Dr. Mark Hatley at St. Jude Children's Research Hospital. The human embryonal kidney cell line 293FT was obtained from Invitrogen. Cells were grown in either RPMI (Rh30, Rh41, Rh4 and Rh5) or DMEM (293FT) with 10% fetal bovine serum, 1% Penicillin/Streptomycin, 10 mM Hepes, 1x MEM non-essential amino acids, and 1 mM Sodium Pyruvate, in a humidified atmosphere of 5% CO_2 at 37°C. The Human Umbilical Vein Endothelial Cell (HUVEC) line was obtained from Lonza Bioscience (Walkersville, MD, USA) and cultured as previously described [9]. Cell lines were authenticated by short tandem repeat profiling and verified to be mycoplasma-free. YK-4-279 was obtained from Selleck Chemicals (#S7679; Houston, TX, USA) as a racemic mixture, and was dissolved in DMSO for experiments.

DNA constructs for protein expression studies

pcDNA3-PAX3-FOXO1/FKHR (plasmid #115526) was obtained from Addgene (Watertown, MA, USA). The expression plasmid pCMV-FLAG-hETS1 was constructed by subcloning FLAG epitope-tagged human ETS1 from the plasmid ETS1_pet28a (Addgene #131663) into the mammalian expression plasmid pcDNA3.1. The expression plasmid pCMV-FLAG-PAX3-FOXO1 was constructed by subcloning PAX3-FOXO1 from pBABE-PAX3-FOXO1 (kind gift of Mark Hatley [10]) into the mammalian expression plasmid pT-FLAG (Addgene #31385). The

expression plasmid pCMV-hETS1 was constructed by subcloning hETS1 from pCMV-FLAG-hETS1 into the expression plasmid pcDNA3.1. All subcloning used standard molecular techniques and was confirmed by Sanger sequencing.

Protein Immunoblotting

Protein immunoblotting was performed as previously described [9]. Primary antibodies used were: ETS1 (Cell Signaling Technologies (CST; Danvers, MA, USA); #14069; 1:1000), FOXO1 (CST; #2880; 1:1000), KDM3A (ProMab Biotechnologies, Richmond, CA, USA; #30134; 1:1000), BRG1 (CST; #49360; 1:1000), PODXL (Santa Cruz Biotechnology, Dallas, TX, USA; #23904; 1:1000), FLAG (Sigma-Aldrich, St. Louis, MO, USA; #F1804; 1:1000) and alpha-Tubulin (Sigma-Aldrich; #T5168; 1:20000).

Quantification of RNA expression

RNA expression quantification was performed as previously described [9]. Cells were harvested at 70-80% confluence in TRIzol (Invitrogen; Waltham, MA, USA), and RNA was extracted per manufacturer instructions. RNA levels of specific transcripts were assessed by qRT-PCR (using qScript Super Mix and Perfecta SYBR Green Fast Mix; Quantabio, Beverly, MA, USA) with RPL19 RNA as internal control (primers are listed in Supplementary Table S1).

Stable depletion of gene expression

Stable shRNA-mediated depletion of gene expression in FP-RMS cells, via lentiviral delivery, was performed as previously described [9]. Scrambled nontargeting control shRNA (Addgene plasmid #1864), and KDM3A and ETS1 targeting shRNAs have been previously validated in the cell lines used here [9]. shRNAs 1 and 2 for PODXL correspond to TRCN 0000296029 and TRCN 0000310117, while shRNAs 1 and 2 for BRG1 correspond to TRCN0000015548 and TRCN0000015549 (all Sigma Mission shRNAs, distributed via the University of Colorado Cancer Center Functional Genomics Shared Resource). Following transduction, cells were selected with 0.5 μg/mL (Rh41), 1 μg/mL (Rh5 and Rh30), or 1.5 μg/mL (Rh4) of puromycin for 3-5 days. Depletion of gene expression was verified using protein immunoblotting.

Growth assays

MTT and clonogenic assays were performed as previously described [9, 11]. Briefly, for MTT assays, 5,000 cells per well were plated in 96-well plates in replicates and growth was determined by optical density following MTT reagent treatment and solubilization. For colony formation assays, 500 cells per well were plated in triplicate in 6-well plates with colonies visualized using crystal violet staining. For drug treatment studies, IC_{50} values were determined via a non-linear regression plot performed using the GraphPad Prism statistical software package.

Cell migration and invasion assays

Cell migration and invasion assays were performed as previously described [1, 9, 12], with the following modifications.

For migration assays, cells were washed with serum-free media, and 20000 cells (Rh30) or 30000 cells (Rh41) were plated in 200 µL of serum-free media in the top chamber of Boyden Chamber well inserts (8 µM pore, #353097; BD Biosciences, Franklin Lakes, NJ, USA) in replicates. Inserts were then placed in a companion 24-well plate with 600 µL of media containing 10% fetal bovine serum as chemoattractant in the bottom chamber. Invasion assays followed the same protocol, but used: 75000 cells (Rh30) or 100000 cells (Rh41); Boyden Chamber inserts with Matrigel (8 µM pore, #354480; Corning Life Sciences, Tewksbury, MA, USA); media containing 5% fetal bovine serum as chemoattractant in the bottom chamber. After 12 hours (for migration) or 24 hours (for invasion), migrated/invaded cells were fixed for 20 min in 70% ethanol. Unmigrated/uninvaded cells and Matrigel were removed by cleaning the top of the membrane with a cotton swab. Migrated/invaded cells were permeabilized for 5 min in 0.3% Triton-X in PBS. Cells were stained with 3 µg/mL DAPI in PBS for 20 min. Five random fields at 10X power were taken of each well using the Nikon Eclipse Ti microscope. Quantification was carried out using the Nikon NLS software object count and ImageJ.

For transendothelial invasion assays, 10000 Human Umbilical Vein Endothelial Cells (HUVEC) were plated in the top of the Boyden Chamber well insert (8 µM pore, BD Biosciences, #353097), and inserts were placed in a 24-well companion plate with 600 µL of EBM media (Lonza, #185299). Following HUVEC attachment and formation of a confluent monolayer (~12 hours), the HUVEC monolayer was washed twice with PBS. Then, the indicated RMS cells were harvested, counted, and stained with Calcein AM dye (ThermoFisher Scientific, Waltham, MA, USA; C1430). The RMS cells were further washed with serum-free media, and 100000 cells (Rh30) or 150000 cells (Rh41) were plated in 200 µL of serum-free media on top of the HUVEC monolayer. The inserts were then placed in a companion 24-well plate with 600 µL of media

containing 5% fetal bovine serum as chemoattractant in the bottom chamber. After 16 hours, uninvaded cells and HUVECs were removed by cleaning the top of the membrane with a cotton swab. Invaded cells were visualized using the Nikon Eclipse Ti microscope, and five random fields at 10X power were imaged. Quantification was carried out using the Nikon NLS software object count and ImageJ.

***In vivo* xenograft studies**

Tail vein injection xenograft model studies were performed as previously described [1, 9]. Briefly, 1 X 10^6 Scramble control or shPODXL Rh30 cells, each additionally expressing a luciferase reporter, were injected into the tail vein of NOD-SCID/Gamma mice. Sample size was determined by our past experience [1, 9, 12-15]. Mice were ~ 3 months in age, male and female; control and experimental groups were matched as equally as possible for sex and age. No further randomization or blinding was performed. Metastasis development was monitored weekly using *in vivo* imaging system (IVIS) visualization following administration of luciferin. All animal experiments were in compliance with ethical regulations as approved by our Institutional Animal Care and Use Committee.

Transcriptome profiling and analysis

Transcriptome profiling was performed on triplicate samples of Rh30 cells treated for 24 hours with 1 µM YK-4-279 or vehicle (DMSO) control. RNA was isolated using TRIzol (Invitrogen) and further purified using the Qiagen MinElute column kit (Qiagen, Germantown, MD, USA), both

following manufacturer instructions. Samples were submitted to Novogene Corporation Inc. (Sacramento, CA, USA) for analysis of RNA quality, library preparation, and paired-end (PE150) mRNA next-generation sequencing on an Illumina platform. The quality of the fastq files was accessed using FastQC (v.0.11.8) [16], FastQ Screen (v.0.13.0) [17] and MultiQC (v.1.8) [18]. Illumina adapters and low-quality reads were filtered out using BBDuk (v. 38.87) (http://jgi.doe.gov/data-and-tools/bb-tools). Trimmed fastqc files were aligned to the hg38 human reference genome and aligned counts per gene were quantified using STAR (v.2.7.9a) [19]. Differential gene expression analysis was performed using the DESeq2 package [20]. Enrichment analysis was performed using GSEA [21].

CUT&RUN

Cleavage Under Targets & Release Using Nuclease (CUT&RUN) cistrome profiling was performed using an adaptation of the EpiCypher CUTANA CUT&RUN protocol (https://www.epicypher.com/resources/protocols/cutana-cut-and-run-protocol/), with additional optimization for FP-RMS cells. At least 500000 cells/sample were harvested using brief (under 5 minutes) 0.25% trypsin treatment. Cells were counted, viability assessed, and washed twice in Wash Buffer (20 mM Hepes pH 7.5, 150 mM NaCl, 0.5 mM Spermidine supplemented with Roche cOmplete EDTA-free protease inhibitor; #11836170001). Cells were immobilized onto activated CUTANA Concanavalin-A paramagnetic beads (EpiCypher, Durham, NC, USA; #21-1401) for 10 min and then incubated with antibodies overnight in the Antibody Buffer (Wash Buffer + 2mM EDTA + 0.015% Digitonin to permeabilize cells) at 4°C at a 1:50 antibody to reaction volume ratio. Antibodies used were: FOXO1 (CST; #2880S), KDM3A (Bethyl Laboratories, Montgomery, TX, USA; #A301-538A), ETS1 (Active Motif, Carlsbad, CA, USA;

#39580), BRG1 (CST; #49360S), and IgG (EpiCypher; #13-0042). After antibody incubation, unbound antibodies were washed away with cold Digitonin Buffer (Wash Buffer + 0.015% Digitonin). pAG-MNase (EpiCypher; #15-1016) was added to each sample for 10 min at RT before washing away unbound pAG-MNase with cold Digitonin Buffer. The reaction was cooled to near 0°C. Then, 1 µL of ice-cold 100 mM $CaCl_2$ was added, and the reaction was incubated for 2 hours at 4°C. The MNase digestion was terminated with the addition of a master mix of STOP Buffer (340 mM NaCl, 20 mM EDTA, 4 mM EGTA, 50 µg/mL RNaseA, 50 µg/mL Glycogen) and 0.5 ng/µL of *E. coli* DNA (Epicypher). This mixture was incubated for 10 min at 37°C. DNA fragments were purified using the NEB Monarch DNA cleanup kit (New England Biolabs, Ipswitch, MA, USA). DNA yield was assessed by a Promega Quantus fluorometer (Promega, Madison, WI, USA). CUT&RUN libraries were assembled using the NEBNext Ultra II DNA Library Prep Kit for Illumina and dual-index primers following manufacturer protocols, with modifications made for CUT&RUN libraries and to enrich for smaller fragments. Briefly, in the 'NEBNext End Prep' step, the samples were run at 50°C for 60 min, instead of 65°C for 30 min. In the 'Cleanup of Adaptor-ligated DNA' step, 1.75X of AMPure XP beads (Beckman Coulter Life Sciences, Indianapolis, IN, USA) was added, instead of 1.1X of AMPure XP beads. Library size distribution was assessed by Agilent 2100 Bioanalyzer instrument with High Sensitivity DNA Chips (Agilent Technologies, Santa Clara, CA, USA). CUT&RUN libraries were pooled and submitted to the University of Colorado Genomics Shared Resource for paired-end (PE150) next-generation sequencing on the Illumina NovaSEQ 6000 instrument.

CUT&RUN data analysis

CUT&RUN data analysis used similar pipelines to those described previously [22, 23]. The quality of the fastq files was assessed using FastQC [16] and MultiQC [18]. Illumina adapters

and low-quality reads were filtered out using BBDuk (https://jgi.doe.gov/data-and-tools/software-tools/bbtools/bb-tools-user-guide/). Bowtie2 (v.2.3.4.3) [24] was used to align the sequencing reads to the hg38 reference human genome. Samtools (v.1.11) [25] was used to select the mapped reads (samtools view -b -q 30) and sort the bam files. PCR duplicates were removed using Picard MarkDuplicates tool (http://broadinstitute.github.io/picard/). For quantitative comparison of control (Scramble shRNA or vehicle) and experimental (targeting shRNA or drug) samples, a normalization ratio for each sample was calculated by dividing the number of uniquely mapped human reads of the sample with the lowest number of reads by the number of uniquely mapped human reads of each sample. These normalization ratios were used to randomly sub-sample reads to obtain the same number of reads for each sample using samtools view -s. Bedtools genomecov was used to create bedgraph files from the bam files [26]. Bigwig files were created using deepTools bamCoverage [27] and visualized using the Integrative Genomics Viewer (IGV) [28]. Peaks were called using MACS2 (v2.1.2) [29], at q<0.1 relative to negative antibody control. Peaks common to both cell lines (Rh30 and Rh41), and factors (KDM3A, ETS1, and/or PAX3-FOXO1) were determined using BedSect (https://imgsb.org/bedsect) [6]. Peak genomic distribution was analyzed using ngs.plot [30]. Cistrome-GO (http://go.cistrome.org) [2] was used to integrate CUT&RUN data with RNAseq data.

Immunoprecipitation

For endogenous protein immunoprecipitation in FP-RMS cells, cells were harvested via 0.25% trypsin treatment, washed with cold phosphate-buffered saline (PBS), and quickly spun down. Cells were resuspended and lysed in cold Co-IP Lysis Buffer (20 mM Tris pH8, 137 mM NaCl, 2 mM EDTA, 1% NP-40) with protease inhibitors (Roche; #11836170001) on ice for 1 hour,

vortexing every 10 minutes. Insoluble material from the whole cell lysates was removed by centrifugation. Lysate protein was quantified via Bradford assay and normalized to the lowest sample amount. 2.5 µg of Rabbit anti-ETS1 antibody (CST; #14069) or the corresponding isotype, species, and amount-matched, Rabbit anti-IgG antibody (CST; #3900) were added to the lysate and incubated at 4°C overnight. As indicated, 100 µg/mL of Ethidium Bromide (Promega; #H5041) or 1 µL/mL of 27.7 U/µL Benzonase (EMD Millipore, Billerica, MA, USA; #70746-3) was also included in the immunoprecipitation reactions to disrupt protein-nucleic acid interactions. Then, the lysates were loaded onto equilibrated Pierce Protein A/G Magnetic Beads (ThermoFisher Scientific; #88802) and incubated for 4 hours at 4°C. The beads were then pelleted via magnet and the supernatant removed. The pellets were washed three times with cold Co-IP Lysis Buffer, resuspending well each time. Lastly, the proteins were eluted off the beads by adding reducing SDS-PAGE buffer (containing 10% beta-mercaptoethanol) and boiling for 8 minutes. Immunoblotting was performed as described above. For immunoprecipitation studies with YK-4-279 treatment, cells were additionally treated with either 1 µM YK-4-279 or vehicle (DMSO) for 16 hours prior to cell lysis, and 1 µM of YK-4-279 or DMSO was included in the immunoprecipitation reactions. Immunoblot detection was performed using ETS1 (CST; #14069; 1:1000) and FOXO1 (CST; #2880; 1:1000) antibodies.

For immunoprecipitation following ectopic expression in 293FT cells, 1.5 µg each of plasmids pCMV-FLAG-hETS1 and pcDNA3-PAX3-FOXO1/FKHR, or pCMV-FLAG-PAX3-FOXO1 and pCMV-hETS1 for the reciprocal experiments, were co-transfected into 293FT cells using TurboFect (ThermoFisher Scientific) following manufacturer protocol. After 48 hours, the transfected 293FT cells were collected for whole cell lysate preparation and immunoprecipitation, each performed as described above. 2.5 µg of Mouse anti-FLAG antibody (Sigma-Aldrich; #F1804) were added for the immunoprecipitation. The corresponding species,

isotype, and amount matched IgG antibody (Mouse anti-IgG antibody [CST; #5415]) was also used in separate immunoprecipitation reactions as a negative control. Immunoblot detection was performed using ETS1 (CST; #14069; 1:1000) and FOXO1 (CST; #2880; 1:1000) antibodies.

Statistical Analysis

GraphPad Prism software was used for all statistical analyses of experimental data. Statistical tests performed, and their interpretation (degrees of statistical significance), are detailed in the respective figure legends.

Supplementary References

1 Sobral LM, Hicks HM, Parrish JK, McCann TS, Hsieh J, Goodspeed A *et al*. KDM3A/Ets1 epigenetic axis contributes to PAX3/FOXO1-driven and independent disease-promoting gene expression in fusion-positive Rhabdomyosarcoma. *Mol Oncol* 2020; 14: 2471-2486.

2 Li S, Wan C, Zheng R, Fan J, Dong X, Meyer CA *et al*. Cistrome-GO: a web server for functional enrichment analysis of transcription factor ChIP-seq peaks. *Nucleic Acids Res* 2019; 47: W206-w211.

3 Cao L, Yu Y, Bilke S, Walker RL, Mayeenuddin LH, Azorsa DO *et al*. Genome-wide identification of PAX3-FKHR binding sites in rhabdomyosarcoma reveals candidate target genes important for development and cancer. *Cancer Res* 2010; 70: 6497-6508.

4 Sunkel BD, Wang M, LaHaye S, Kelly BJ, Fitch JR, Barr FG *et al*. Evidence of pioneer factor activity of an oncogenic fusion transcription factor. *iScience* 2021; 24: 102867.

5 Gryder BE, Yohe ME, Chou HC, Zhang X, Marques J, Wachtel M *et al*. PAX3-FOXO1 Establishes Myogenic Super Enhancers and Confers BET Bromodomain Vulnerability. *Cancer Discov* 2017; 7: 884-899.

6 Mishra GP, Ghosh A, Jha A, Raghav SK. BedSect: An Integrated Web Server Application to Perform Intersection, Visualization, and Functional Annotation of Genomic Regions From Multiple Datasets. *Front Genet* 2020; 11: 3.

7 Laubscher D, Gryder BE, Sunkel BD, Andresson T, Wachtel M, Das S *et al*. BAF complexes drive proliferation and block myogenic differentiation in fusion-positive rhabdomyosarcoma. *Nat Commun* 2021; 12: 6924.

8 Almacellas-Rabaiget O, Monaco P, Huertas-Martinez J, Garcia-Monclus S, Chicon-Bosch M, Maqueda-Marcos S *et al*. LOXL2 promotes oncogenic progression in alveolar rhabdomyosarcoma independently of its catalytic activity. *Cancer Lett* 2020; 474: 1-14.

9 Sobral LM, Sechler M, Parrish JK, McCann TS, Jones KL, Black JC *et al*. KDM3A/Ets1/MCAM axis promotes growth and metastatic properties in Rhabdomyosarcoma. *Genes Cancer* 2020; 11: 53-65.

10 Hanna JA, Garcia MR, Lardennois A, Leavey PJ, Maglic D, Fagnan A *et al*. PAX3-FOXO1 drives miR-486-5p and represses miR-221 contributing to pathogenesis of alveolar rhabdomyosarcoma. *Oncogene* 2018; 37: 1991-2007.

11 Moore C, Parrish JK, Jedlicka P. MiR-193b, downregulated in Ewing Sarcoma, targets the ErbB4 oncogene to inhibit anchorage-independent growth. *PLoS One* 2017; 12: e0178028.

12 Sechler M, Parrish JK, Birks DK, Jedlicka P. The histone demethylase KDM3A, and its downstream target MCAM, promote Ewing Sarcoma cell migration and metastasis. *Oncogene* 2017; 36: 4150-4160.

13 McCann TS, Parrish JK, Hsieh J, Sechler M, Sobral LM, Self C *et al*. KDM5A and PHF2 positively control expression of pro-metastatic genes repressed by EWS/Fli1, and promote growth and metastatic properties in Ewing sarcoma. *Oncotarget* 2020; 11: 3818-3831.

14 Parrish JK, McCann TS, Sechler M, Sobral LM, Ren W, Jones KL *et al*. The Jumonji-domain histone demethylase inhibitor JIB-04 deregulates oncogenic programs and increases DNA damage in Ewing Sarcoma, resulting in impaired cell proliferation and survival, and reduced tumor growth. *Oncotarget* 2018; 9: 33110-33123.

15 Parrish JK, Sechler M, Winn RA, Jedlicka P. The histone demethylase KDM3A is a microRNA-22-regulated tumor promoter in Ewing Sarcoma. *Oncogene* 2015; 34: 257-262.

16 Andrews S. FastQC: A Quality Control Tool for High Throughput Sequence Data. *Online* 2010.

17 Wingett SW, Andrews S. FastQ Screen: A tool for multi-genome mapping and quality control. *F1000Res* 2018; 7: 1338.

18 Ewels P, Magnusson M, Lundin S, Käller M. MultiQC: summarize analysis results for multiple tools and samples in a single report. *Bioinformatics* 2016; 32: 3047-3048.

19 Dobin A, Davis CA, Schlesinger F, Drenkow J, Zaleski C, Jha S *et al*. STAR: ultrafast universal RNA-seq aligner. *Bioinformatics* 2013; 29: 15-21.

20 Love MI, Huber W, Anders S. Moderated estimation of fold change and dispersion for RNA-seq data with DESeq2. *Genome Biol* 2014; 15: 550.

21 Zhu A, Srivastava A, Ibrahim JG, Patro R, Love MI. Nonparametric expression analysis using inferential replicate counts. *Nucleic Acids Res* 2019; 47: e105.

22 Hsu JY, Danis EP, Nance S, O'Brien JH, Gustafson AL, Wessells VM *et al*. SIX1 reprograms myogenic transcription factors to maintain the rhabdomyosarcoma undifferentiated state. *Cell Rep* 2022; 38: 110323.

23 Hughes CJ, Fields KM, Danis EP, Hsu JY, Neelakantan D, Vincent MY *et al*. SIX1 and EWS/FLI1 co-regulate an anti-metastatic gene network in Ewing Sarcoma. *Nat Commun* 2023; 14: 4357.

24 Langmead B, Salzberg SL. Fast gapped-read alignment with Bowtie 2. *Nature Methods* 2012; 9: 357-359.

25 Li H, Handsaker B, Wysoker A, Fennell T, Ruan J, Homer N *et al*. The Sequence Alignment/Map format and SAMtools. *Bioinformatics* 2009; 25: 2078-2079.

26 Quinlan AR, Hall IM. BEDTools: a flexible suite of utilities for comparing genomic features. *Bioinformatics* 2010; 26: 841-842.

27 Ramírez F, Ryan DP, Grüning B, Bhardwaj V, Kilpert F, Richter AS *et al*. deepTools2: a next generation web server for deep-sequencing data analysis. *Nucleic Acids Res* 2016; 44: W160-165.

28 Robinson JT, Thorvaldsdóttir H, Winckler W, Guttman M, Lander ES, Getz G *et al*. Integrative genomics viewer. *Nature Biotechnology* 2011; 29: 24-26.

29 Zhang Y, Liu T, Meyer CA, Eeckhoute J, Johnson DS, Bernstein BE *et al*. Model-based Analysis of ChIP-Seq (MACS). *Genome Biology* 2008; 9: R137.

30 Shen L, Shao N, Liu X, Nestler E. ngs.plot: Quick mining and visualization of next-generation sequencing data by integrating genomic databases. *BMC Genomics* 2014; 15: 284.
